# *Salmonella* Typhimurium vitamin B12-dependent methionine metabolism regulates *Caenorhabditis elegans* development and vitellogenesis

**DOI:** 10.1128/spectrum.00827-26

**Published:** 2026-09-29

**Authors:** Chamjailiu Daimai, Vidya Devi Negi

**Affiliations:** 1 Department of Biological Sciences, Indian Institute of Science Education and Research (IISER) Mohali, SAS Nagar, Punjab, India; Universidad Andres Bello, Santiago, Chile

**Keywords:** *Salmonella*, development, B12 vitamin, metabolism, *C. elegans*

## Abstract

Host-microbe interactions extend beyond infection, involving microbial metabolites, such as short-chain fatty acids, vitamins, and other bioactive compounds, that influence host physiology and development. Using *Caenorhabditis elegans*, an excellent model organism for host-microbe interactions, we demonstrate that *Salmonella* Typhimurium significantly accelerates *C. elegans* growth through a vitamin B12-dependent mechanism affecting methionine metabolism. We measured the reproductive maturity of *C. elegans* via embryo and oocyte production in response to various *Salmonella* virulence factor mutants and *Salmonella* strains deficient in cobalamin, folate, and methionine synthesis pathways. We found that the *Salmonella cbiB* and *metH* genes are essential for rapid development in *C. elegans*. Moreover, the *C. elegans metr-1* mutant, which lacks functional methionine synthase, did not exhibit increased reproductive maturity when fed on *S*. Typhimurium or *Escherichia coli* OP50 supplemented with vitamin B12. However, methionine supplementation rescued this phenotype, indicating that *C. elegans* obtains vitamin B12 from *S*. Typhimurium, enhancing the methionine/S-adenosylmethionine (MET/SAM) cycle. We further observed that the *C. elegans sams-1* mutant, deficient in SAM synthase, showed growth defects and reduced oocyte production when fed on *Salmonella* or OP50 supplemented with vitamin B12 or methionine, respectively. Thus, SAM emerges as a key link between bacterial vitamin B12 metabolism and host reproduction. Additionally, *Salmonella* increases vitellogenin-2 expression, reduces glutathione S-transferase activity, and decreases mitochondrial function. However, Δ*cbiB* and Δ*metH* reversed the beneficial effects of vitamin B12-dependent methionine on vitellogenesis and oxidative stress, suggesting that *Salmonella* promotes vitellogenesis and reduces oxidative stress through vitamin B12-dependent mitochondrial regulation, linking microbial metabolism to host development.

**IMPORTANCE:**

Understanding the interactions between hosts and pathogens is essential for improving host health. *Caenorhabditis elegans* obtains micronutrients from diet, gut microbiota, and supplements, but the contribution of microbiota in nutrient absorption is not fully understood. Bacterial metabolism may influence micronutrient status and pathogenicity, resulting in both positive and adverse health effects on the host. This study demonstrates that pathogenic *Salmonella* can serve as a nutritional source for *C. elegans* when the two organisms coexist in the same environment. The findings suggest that, rather than being solely detrimental, *Salmonella* can alter the metabolism of *C. elegans*, highlighting a complex interaction between the pathogen and its host. This relationship may provide insights into the ecological dynamics of microbial communities and their influence on host physiology and development.

## INTRODUCTION

Host-pathogen interactions are complex and involve multiple regulatory mechanisms. The interplay between a host response and pathogen virulence is intricate (1, 2). Pathogens compete with the host for nutrients, altering infection outcomes (3). Host metabolism shifts to secure essential resources during infection, driven by immune activation and pathogen strategies (4). Consequently, host metabolic pathways adapt to stress and reallocate nutrients to meet physiological needs during infection (5). *Caenorhabditis elegans (C. elegans*) encounters various bacteria in its natural habitat, including pathogens (6, 7). *C. elegans* has emerged as a valuable model for studying immunometabolism (8). *C. elegans* has a 3-day lifecycle progressing through embryogenesis, four larval stages (L1–L4), and adulthood (9, 10). Host-pathogen interactions affect reproductive physiology, but few studies have investigated how pathogens influence this physiology (11, 12). *Salmonella* Typhimurium also causes major gastrointestinal and systemic diseases in humans and animals (13). The pathogenesis of *Salmonella* is primarily regulated by the virulence plasmid (pSLT), particularly through the *spv* operon, secretion systems, and *Salmonella* Pathogenicity Islands (SPIs) (14, 15). *Salmonella* infection accelerates larval development, reduces lifespan, and induces developmental changes linked to host pathways and *Salmonella* virulence factors, highlighting how bacterial pathogens can modulate *C. elegans* development (16). Using *C. elegans*, we investigated how *Salmonella* Typhimurium (*S*. Typhimurium) affects host reproduction through vitamin B12-dependent methionine metabolism (5).

*C. elegans* obtains B vitamins, folate, vitamin B12, vitamin B6, and iron through dietary intake from bacteria and bacterial metabolism in the gut microbiota (17). Vitamin B12 is a key micronutrient produced only by certain prokaryotes but is needed by various animals, including *C. elegans* (18, 19). Vitamin B12 is an essential cofactor in two key metabolic pathways. It aids methylmalonyl-CoA mutase (MUT, MMCM-1 in *C. elegans*) in mitochondrial propionate metabolism. It supports methionine synthase (MS, METR-1 in *C. elegans*) in one-carbon metabolism, including the methionine and folate cycles (20, 21). Vitamin B12 deficiency is associated with diverse clinical pathologies, including anemia, neuropathy, and congenital disabilities (22). In *C. elegans*, it leads to infertility, slower growth rate, and reduced lifespan (23). *Escherichia coli (E. coli*) cannot synthesize vitamin B12 *de novo* and solely produces vitamin B12 through the salvage pathway. Hence, the levels of vitamin B12 in *E. coli* are significantly lower compared to species such as *Salmonella* and *Comamonas*, which can synthesize it *de novo*. The methionine biosynthetic pathway, highly conserved across prokaryotes, is absent in vertebrates, making it a plausible antimicrobial target (24). However, *de novo* methionine biosynthesis is essential in *S*. Typhimurium (13, 25). Methionine is crucial for protein synthesis and S-adenosylmethionine (SAM) production, supporting methylation and animal reproductive output (26, 27). It influences the expression of the vitellogenin gene, affecting yolk production and reproductive fitness. Consequently, the availability of methionine directly impacts vitellogenesis and embryo development in *C. elegans* and other species (28–30). In addition to yolk formation, vitellogenins in *C. elegans* also enhance the host’s defense against pathogens (31). Exposure to certain bacteria accelerates reproductive maturity without impacting total reproduction (32, 33). Notably, methionine metabolism plays a crucial role in balancing oxidative stress by maintaining mitochondrial integrity, providing cysteine for glutathione synthesis, and facilitating methylation reactions that regulate antioxidant defenses (34, 35). Optimal reproductive success depends on the coordinated activity of methionine metabolism, efficient antioxidant defense, and healthy mitochondrial function. Disruption in any component can lead to impaired fertility, defective oocyte provisioning, or increased embryonic mortality, thereby linking metabolic health to reproduction (36, 37). Our findings show that the *cbiB* and *metH* genes in *Salmonella* are vital for regulating the MET/SAM cycle *in C. elegans*, thereby enhancing reproductive maturity. We hypothesized that *S*. Typhimurium’s ability to synthesize cobalamin may accelerate *C. elegans* development through vitamin B12-dependent methionine metabolism, rather than through pathogenic effects. We investigated this by analyzing *Salmonella* mutants lacking cobalamin and methionine biosynthesis, as well as *C. elegans* mutants deficient in vitamin B12 pathways. Our findings indicate that *S*. Typhimurium improves development and oocyte production in *C. elegans* primarily by increasing dietary vitamin B12, which in turn increases methionine and SAM synthesis, leading to increased vitellogenin production and better mitochondrial health. This study reveals a metabolic relationship between a pathogen and its host, underlining the role of microbial metabolism in nematode reproduction and developmental regulation.

## RESULTS

### *Caenorhabditis elegans* development alteration is independent of *Salmonella* virulence

Previously, the lab has shown how *S*. Typhimurium (WT-STM) infection of *C. elegans* leads to rapid development compared to *E. coli* OP50 (OP50) (16). In the current study, we exposed *C. elegans* to WT-STM and several attenuated *Salmonella* mutant strains, including Δ*hilA*, Δ*avrA*, Δ*sptP*, Δ*sipD*, Δ*ssaV*, Δ*ygiM*, Δ*rspA*, Δ*sopB*, Δ*mgtC*, Δ*phoP*, and Δ*fepB*, to understand the role of major virulence factors in regulating *C. elegans* development. Most of these mutant strains are avirulent or attenuated deletion strains, listed in Table 1 (38–45). *C. elegans* embryo production was quantified at 56 h post-L1 stage to observe the developmental dynamics upon infection with these strains. Interestingly, the mutants showed accelerated development compared to *E. coli* OP50, with embryo production comparable to that of WT-STM (Fig. 1A). The number of oocytes produced was also quantified in day 1 adult *C. elegans*. A similar outcome was observed, with increased oocyte production during infection with the WT-STM and its avirulent mutant strains (Fig. 1B). We aimed to identify signaling pathways involved in WT-STM infection and its mutants’ mediated enhanced development by analyzing gene expression of insulin/IGF-1 signaling, the RAS/MAPK pathway, and programmed cell death associated with oogenesis in *C. elegans* (46, 47). The pathway is initiated by the binding of LIN-3 (EGF-like ligand) to the LET-23 receptor tyrosine kinase on the surface of vulva precursor cells. A kinase cascade is activated with the downstream kinase MPK-1, which is activated in the pachytene and oocyte regions of the germline (48, 49). PAR-5 is a negative regulator of MPK-1 signaling, restricting its activity in the proximal region (50). In contrast, GLP-1/Notch signaling in the distal germline promotes germline stem cell proliferation and prevents premature entry into meiosis (51–53). IGF-1 is involved in various processes, including growth, metabolism, and lifespan, and it is activated when insulin-like peptides bind to the DAF-2 receptor, initiating a signaling cascade (54). Additionally, CED-9 inhibits CED-4, and upon binding to EGL-1, it initiates programmed cell death by releasing CED-4, which then oligomerizes with the active caspase CED-3 to execute cell death (55, 56). However, the mRNA expression levels of these major target genes, such as *lin-3*, *let-23*, *mpk-1*, *glp-1*, *par-5*, *ced-9*, *ced-4*, *ced-3*, *daf-2*, and *ins-7*, suggest that they were not responsible for the rapid reproductive maturity observed during *Salmonella* infection (Fig. S1A through C). There was no increase in the lifespan of *C. elegans* exposed to various major SPI-1 effector mutants (Fig. S1D) as compared to WT-STM. This indicates that *C. elegans* exposed to WT-STM and its avirulent mutant strains attain early reproductive maturity, suggesting that these genes do not contribute to the enhanced development of *C. elegans*. These observations suggest that *S*. Typhimurium’s virulence factors do not contribute to the accelerated development of *C. elegans*. Instead, the presence of these bacteria in the diet may significantly affect the host’s development rather than its virulence.

**TABLE 1:** SPI-1, SPI-2, non-SPI-1, cobalamin, folate, and methionine biosynthesis single-gene deletion *Salmonella* mutants on developmental rate

Category	Genes	Description	Developmental rate
SPI-1	Δ*hilA*-STM Δ*avrA-*STM Δ*sptP*-STM Δ*sipD-*STM Δ*sopB*-STM	A transcriptional activator that regulates SPI-1 gene expression, which is essential for *Salmonella’s* host cell invasion (44) The virulence-associated gene is located within SPI-1; the SPI-1 effector protein (57) *Salmonella* protein tyrosine phosphatase (SptP) is directly responsible for the reversal of the actin cytoskeletal changes induced by other effectors of *Salmonella* via regulating villin phosphorylation (14) One of the four *Salmonella* invasion proteins (Sips) is Sips A–D. These Sips are exported and translocated into the host cell plasma membrane or cytosol and play essential and complex roles in the secretion and translocation of SPI-1 effectors (42) The *Salmonella* outer protein B, an effector protein, mediates virulence by interfering with inositol phosphate signaling pathways and inducing Akt activation (42)	Increased Increased Increased Increased Increased
SPI-2	Δ*ssaV*-STM	*ssaV* is a structural gene encoding part of the secretion apparatus (58)	Increased
Non-SPI	Δ*ygiM-*STM Δ*rspA-*STM Δ*mgtC*-STM Δ*phoP*-STM Δ*fepB*-STM	It is proposed to have an Src homology or SH3 domain, but its function is under investigation in the lab Biofilm formation and virulence A virulence factor crucial for intramacrophage survival and growth in magnesium-depleted environments (38) Forms a two-component regulatory system (PhoP/PhoQ) that controls virulence and adaptation to low magnesium environments (39) Encodes a periplasmic protein crucial for ferric enterobactin uptake, an essential part of the FepBDGC ABC transporter system that helps the bacteria acquire iron, a vital nutrient for survival and virulence (43)	Increased Increased Increased Increased Increased
Cobalamin biosynthesis	Δ*cobB*-STM Δ*cobC*-STM Δ*cobD*-STM Δ*cobS*-STM Δ*cbiB*-STM	NAD-dependent protein deacylase (59) Phosphatase catalyzes the dephosphorylation of α-ribazole-5′-phosphate to α-ribazole and adenosylcobalamin-5′-phosphate to adenosylcobalamin (59) A decarboxylase that converts L-threonine-O-3-phosphate to (R)-1-amino-2-propanol-O-2-phosphate (59) Cobalamin synthetase is required in the final step (59) Encodes an integral membrane protein, adenosylcobinamide-phosphate synthase (59)	Increased Increased Increased Increased Decreased
Folate biosynthesis	Δ*pabA*-STM	Crucial for the biosynthesis of para-aminobenzoic acid (PABA), a precursor for folate synthesis, which is essential for bacterial growth and virulence (59)	Increased
Methionine biosynthesis	Δ*metC*-STM Δ*metE*-STM Δ*metH*-STM	Cystathionine β-lyase catalyzes cystathionine to homocysteine (24) Encodes 5-methyltetrahydropteroyl triglutamate-homocysteine S-methyltransferase, essential for growth and virulence (24) Encodes a cobalamin-dependent enzyme, N(5)-methyltetrahydrofolate-homocysteine transmethylase (24)	Increased Decreased Decreased

**Fig 1:**
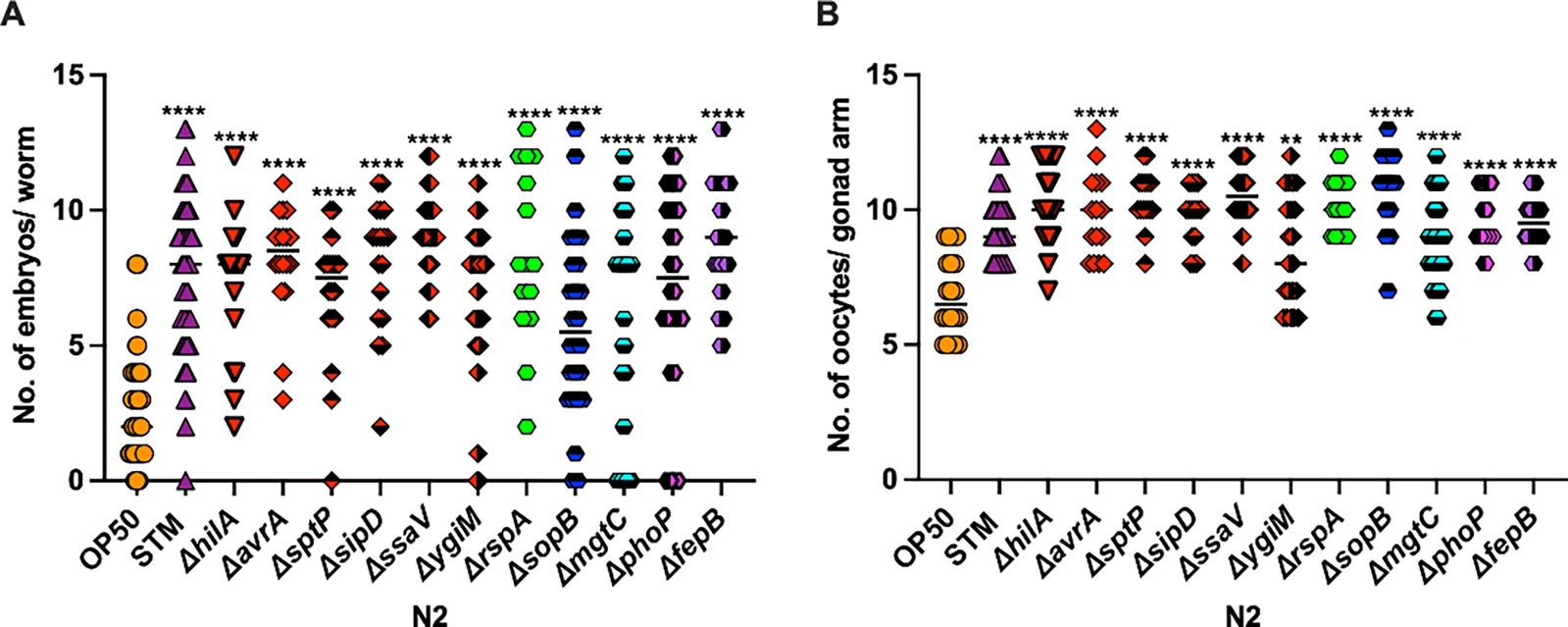
*Caenorhabditis elegans* development alteration is independent of *Salmonella* virulence. (**A**) Represents the quantification of embryos in N2 worms 56 h post-L1 stage and (**B**) oocyte production 72 h post-L1 stage, which increases in WT-STM, ∆*hilA*, ∆*avrA*, ∆*sptP*, ∆*sipD*, ∆*ssaV*, ∆*ygiM*, ∆*rspA*, ∆*sopB*, ∆*mgtC*, ∆*phoP*, and ∆*fepB*-exposed animals. An ordinary one-way ANOVA and a post hoc Tukey HSD test were performed. ****P < 0.0001; ***P < 0.001; **P < 0.01; *P < 0.05.

### *Salmonella* Typhimurium, a vitamin B12-rich diet, accelerates development and oocyte production in *C. elegans*

*Comamonas aquatica* DA1877 is known to accelerate *C. elegans* development (60) via the production of vitamin B12, a crucial cofactor in one-carbon metabolism. Animals acquire vitamin B12 either through a symbiotic relationship with gut bacteria or through their diet. Although *E. coli* can import vitamin B12 from standard growth media, the concentrations are significantly lower than those found in vitamin B12-producing bacteria, such as *Comamonas aquatica* (*C. aquatica*) (20). Unlike *Salmonella* and *Comamonas*, which can synthesize cobalamin *de novo*, *E. coli* lacks this capability and relies solely on the salvage pathway, resulting in a significantly lower intracellular vitamin B12 level (59). To investigate the role of bacterial metabolism in host reproduction, we quantified the number of progenies produced by the *C. elegans* exposed to *E. coli* OP50, WT-STM, Δ*hilA*, and *E. coli* OP50 supplemented with vitamin B12. The Δ*hilA* strain, a SPI-1 master regulator mutant, was used as a control to exclude the pathogenic effects on host reproduction. The number of progenies produced by the worm exposed to WT-STM, Δ*hilA*, and *E. coli* OP50 supplemented with vitamin B12 was increased significantly on the first 2 days; however, by day 3, the number decreased and was comparable to the OP50, likely due to the depletion of the self-fertilized sperm as single hermaphrodites are being transferred to the assayed plates (Fig. 2A). We next examined the oocyte production in day 1 adult *C. elegans* using the OD95, a *pie-1p::mCherry::his-58 + unc-119(+)* reporter strain. We observed a significant increase in oocyte production in WT-STM, Δ*hilA*, and *E. coli* OP50 supplemented with vitamin B12 (Fig. 2B and C). This suggested that vitamin B12, rather than the bacteria’s virulence factor, contributed to the observed enhanced development. Additionally, to directly investigate the role of one-carbon metabolism (OCM), we generated *Salmonella* mutants that are deficient in folate (Δ*pabA*), cobalamin (Δ*cobB*, Δ*cobC*, Δ*cobD*, Δ*cobS*, and Δ*cbiB*), and methionine (Δ*metC*, Δ*metE*, and Δ*metH*) synthesis. In *Salmonella enterica*, folate-dependent OCM is essential for purines, thymidylate, and methionine biosynthesis, as well as for redox balance and translation regulation (61). We also used the VL749 strain, a transgenic worm that carries an *acdh-1::GFP* reporter, thereby enabling detection of vitamin B12 in the environment (20, 62). The *acdh-1* promoter acts as a sensitive sensor that responds specifically to activation of a backup propionate degradation pathway when the canonical B12-dependent pathways are disrupted (40, 41). A reduction in GFP fluorescence indicates the presence of vitamin B12. The worms were exposed to *E. coli* OP50, WT-STM, and *E. coli* OP50 supplemented with vitamin B12, as well as *Salmonella* mutants Δ*cobB*, Δ*cobC*, Δ*cobD*, Δ*cobS*, Δ*cbiB*, and Δ*pabA*. Our findings showed that ACDH-1::GFP expression was significantly reduced in the worm fed with *Salmonella* compared to those fed with *E. coli* OP50 and was almost absent in *E. coli* OP50 supplemented with vitamin B12 (Fig. 2D). We observed a restoration of GFP levels in animals fed with the cobalamin mutants, such as Δ*cobC*, Δ*cobD*, Δ*cobS*, and Δ*cbiB*. However, Δ*cobB-* and Δ*pabA*-exposed VL749 worms showed comparatively lower GFP levels than those of *E. coli* OP50, as the B12 level was not compromised, unlike in other mutants. WT-*Salmonella* effectively silences the reporter, whereas cobalamin-biosynthesis mutants, such as Δ*cobC*, Δ*cobD*, Δ*cobS*, and Δ*cbiB*, fail to suppress GFP expression despite sharing the same metabolic context as the wild type, except for deficiencies in cobalamin. Furthermore, supplementation with exogenous vitamin B12 on *E. coli* OP50 completely abolished the *Pacdh-1::GFP* expression, confirming that it is the influx of intestinal cobalamin, rather than nonspecific dietary adaptations, that drives the observed shifts in gene expression and phenotype (Fig. 2D). These results suggest that *C. elegans* obtains vitamin B12 from its bacterial diet, and the data clearly show its response to the level of B12 synthesized by *Salmonella*.

**Fig 2:**
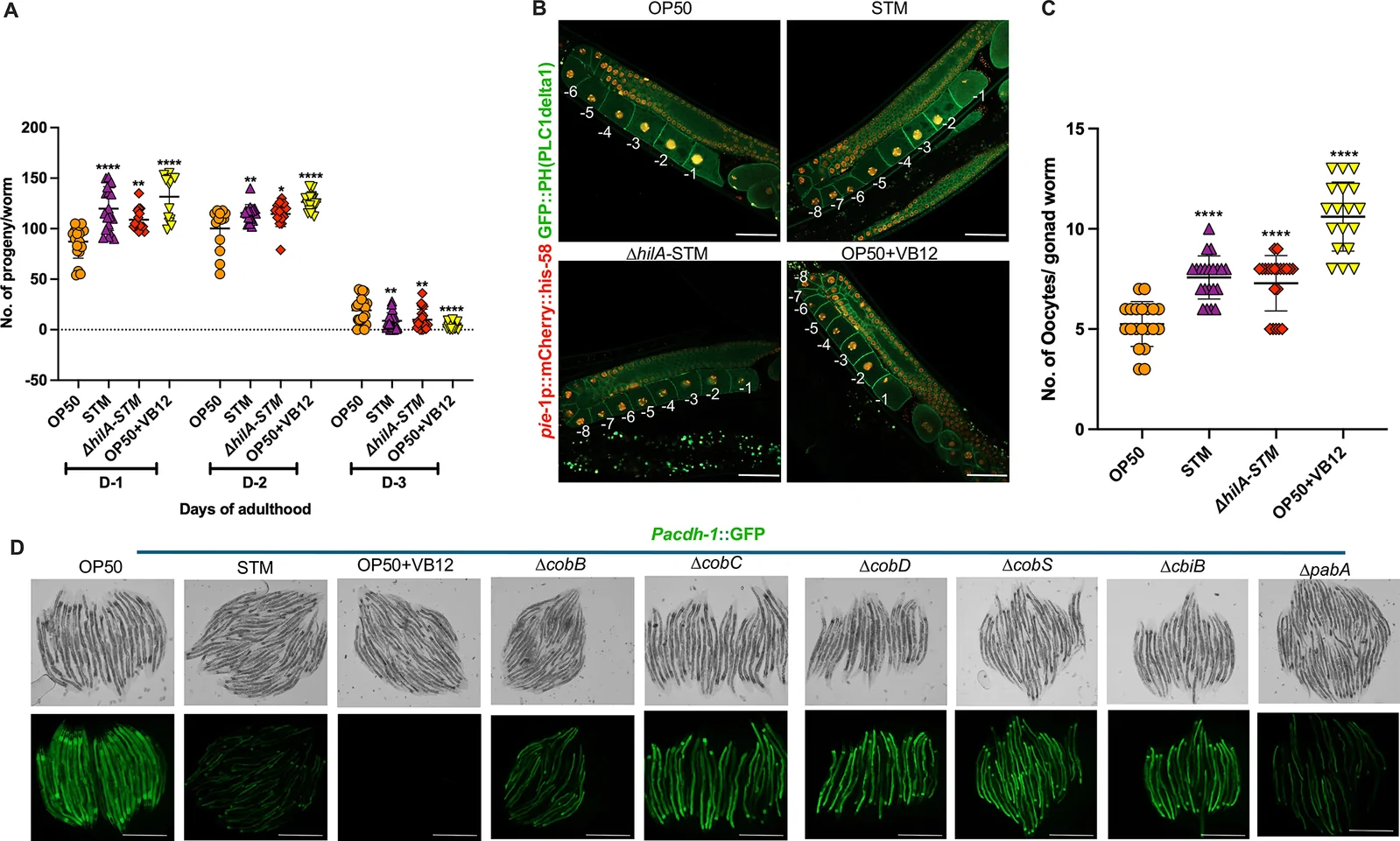
*S*. Typhimurium, a vitamin B12-rich diet accelerates reproductive maturity *in C. elegans.* (**A**) Wild-type *S.* Typhimurium, ∆*hilA-*STM, and vitamin B12-supplemented with *E. coli* OP50 increase the number of progeny within the first 2 days. (**B**) Representative confocal microscopy images of the gonad, using the OD95 (*ltIs37 [pie-1p::mCherry::his-58 + unc-119(+)] IV. ltIs38 [pie-1p::GFP::PH(PLC1delta1) + unc-119(+)]*) transgenic strain, showing increased oocyte production in WT-STM, ∆*hilA-*STM, and OP50 supplementation with vitamin B12-fed day 1 worms. The numbers −1 and −2 indicate the most proximal mature oocytes. (**C**) Graphical data illustrating increased oocyte production in WT-STM, ∆*hilA-*STM, and OP50 supplementation with vitamin B12-fed worms. (**D**) Representative fluorescence images of *acdh-1p::GFP* animals grown on *E. coli* OP50, wild-type *Salmonella* Typhimurium (WT-STM), cobalamin (Δ*cobB*, Δ*cobC*, Δ*cobD*, Δ*cobS*, Δ*cbiB*), and folate synthesis (Δ*pabA*) *Salmonella* mutants, and *E. coli* OP50 supplemented with vitamin B12, where GFP signals were imaged. An ordinary one-way ANOVA and a post hoc Tukey HSD test were performed. ****P < 0.0001; ***P < 0.001; **P < 0.01; *P < 0.05*.* Scale bar = 100 µm.

### *Salmonella* cobalamin (*cbiB*) and methionine (*metH*) synthesis mutants reduce the developmental rate of *C. elegans*

Given the essential role of one-carbon metabolism in *Salmonella* in the synthesis of nucleotides, amino acids, and cofactors, we sought to determine whether disrupting this pathway could mitigate the accelerated reproductive maturity observed in *C. elegans*. Aged-synchronized L1 larvae were fed on *E. coli* OP50, WT-STM, *E. coli* OP50 supplemented with vitamin B12, Δ*cobB*, Δ*cobC*, Δ*cobD*, Δ*cobS*, Δ*cbiB*, *E. coli* OP50 supplemented with methionine, Δ*metC*, Δ*metE*, and Δ*metH*. Embryo production was quantified at 56 h post-L1 stage to assess developmental acceleration. Interestingly, the Δ*cbiB* strain exhibited reduced embryo production, but this was restored by vitamin B12 supplementation (Fig. 3A). Similarly, *C. elegans* fed with Δ*cobB*, Δ*cobC*, Δ*cobD*, Δ*cobS*, and Δ*cbiB* produced fewer oocytes. This phenotype was reversed by vitamin B12 supplementation (Fig. 3B). The animals fed with Δ*metE* and Δ*metH* showed reduced embryo production, which was reversed with methionine supplementation (Fig. 3C). Consistently, the worms fed with Δ*metC*, Δ*metE*, and Δ*metH* produced fewer oocytes. Phenotype was reversed with methionine supplementation (Fig. 3D). Together, these findings demonstrate that the *Salmonella* one-carbon metabolism pathway is crucial in regulating the reproductive maturity of *C. elegans*. Importantly, no cobalamin, folate, or methionine synthesis mutants of *Salmonella* showed growth defects in Luria-Bertani (LB) medium (Fig. 4A and B). To ensure that the developmental differences observed in *C. elegans* were driven explicitly by structural alterations in bacterial vitamin B12 metabolism rather than physical variations in food availability or biomass, we systematically quantified bacterial lawn growth directly on nematode growth medium (NGM) plates. Prior to worm introduction, NGM plates were seeded with a starting bacterial culture adjusted to an OD600 of 0.3. Following a 12-h incubation period, the resulting bacterial lawns were harvested and assessed for total optical density and viable cell counts. Consistent with our rich-media growth profiles, the Δ*cbiB* and Δ*metH* strains exhibited identical growth characteristics to the wild-type *Salmonella* strain on NGM plates, showing similar OD600 readings and absolute colony-forming unit counts (Fig. 4C and D). These data rigorously exclude biomass abundance or differential plate growth for the mutants as potential experimental confounders, confirming that the developmental acceleration phenotype is strictly linked to the functionality of the *cbiB* and *metH* genes. To directly rule out differences in ingestion, feeding rate, or bacterial survival within the host, we quantified *in vivo* bacterial gut colonization by measuring internal colony-forming units per worm at the D1 adult stage. As shown in Fig. 4E, the internal bacterial load of worms fed with the mutants showed no statistically significant difference from that of worms fed with the WT-STM. To verify whether the observed host phenotypes were modulated by condition-dependent pathogenicity or transient expression of virulence machinery, we further performed qRT-PCR in *vivo* from *Salmonella* harvested directly from the *C. elegans* at L4 and D1 stages of the worms and compared with the expression of the target genes in the *in vitro* LB broth and F-media grown cultures of the WT-STM, Δ*cbiB*, and Δ*metH* strains, which mimic SPI-1 and SPI-2 growth conditions, respectively. Transcriptional profiling of the targeted key regulatory cascades controlling SPI-1 and SPI-2 shows that the core SPI-1 master regulator gene*s*, *hilA* and *hilD*, along with the critical structural component, the *invA* gene, remained strictly at baseline in all the strains, uninduced levels during early host development at the L4 stage (Fig. 4F). This establishes that canonical SPI-1 invasion pathways are not actively engaged within the specific developmental window where host reproductive acceleration occurs. Furthermore, analysis of the SPI-2 regulatory system at the D1 adult stage revealed a distinct transcriptional uncoupling rather than global activation of virulence pathways (Fig. 4G). Specifically, expression of the core virulence sensor kinase *ssrA* remained basal and comparable to F-media growth conditions. Conversely, the downstream response regulator *ssrB* was highly upregulated while its structural gene *ssaJ* was downregulated (Fig. 4G). The master biofilm regulator gene, *csgD*, was concurrently upregulated (Fig. 4H). Taken together, these *in vivo* transcriptional measurements directly confirm that host developmental changes are not a consequence of canonical virulence pathway activation under the indicated bacterial conditions, but rather point toward a non-invasive, colonizing physiological state.

**Fig 3:**
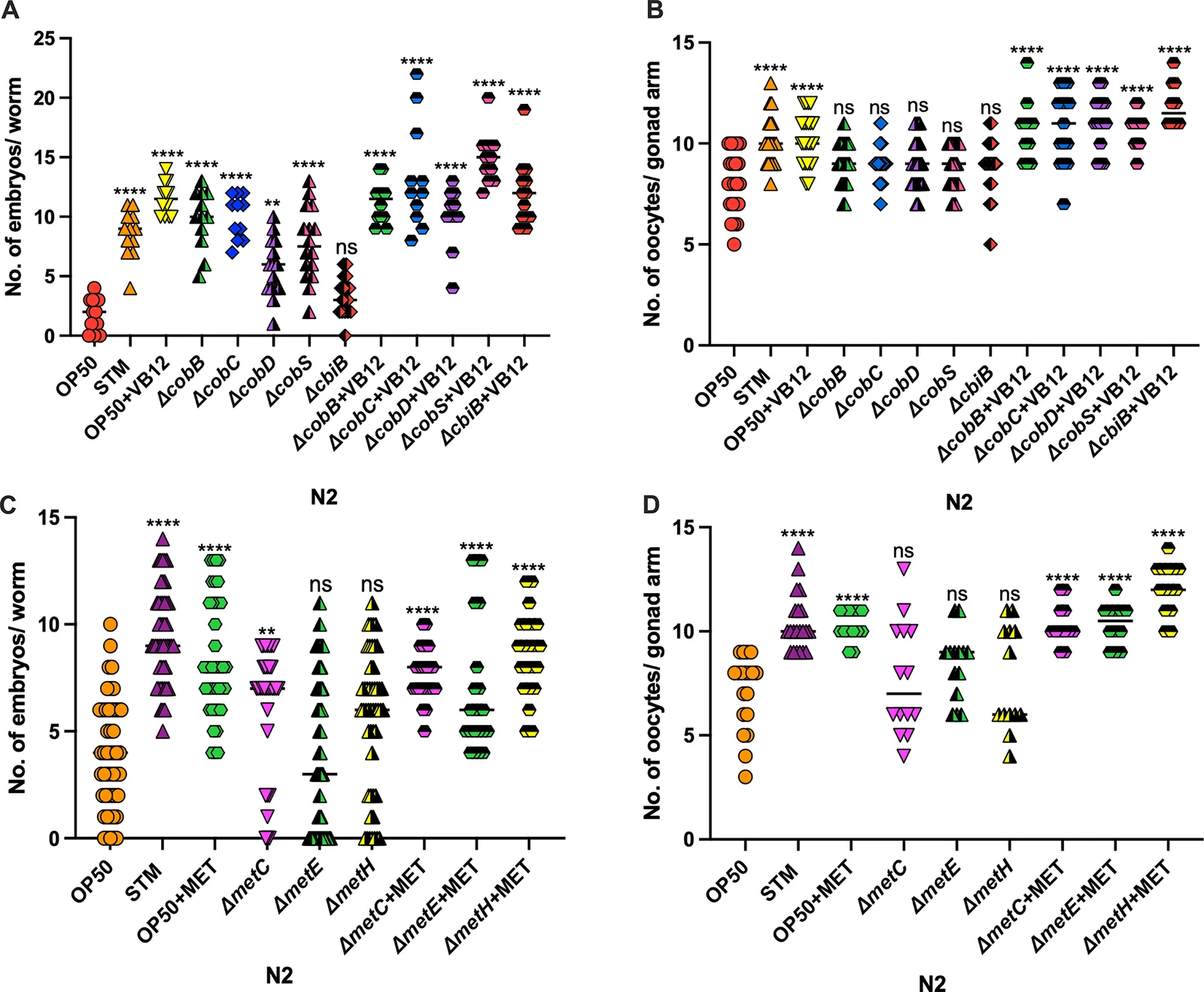
*Salmonella* mutants deficient in cobalamin (*cbiB*) and methionine (*metH*) synthesis reduce the developmental rate of *C. elegans.* Panel **A** represents the quantification of embryos in N2 worms 56 h post-L1 stage, fed on *E. coli* OP50, WT-STM, *E. coli* OP50 supplemented with vitamin B12, Δ*cobB*, Δ*cobC*, Δ*cobD*, Δ*cobS*, Δ*cbiB*, vitamin B12 supplemented with Δ*cobB*, Δ*cobC*, Δ*cobD*, Δ*cobS*, and Δ*cbiB*, respectively. *cbiB Salmonella* had lower embryo production at 56 h post-L1, and supplementation with vitamin B12 increased the embryo number. Panel **B** represents the quantification of oocyte production in N2 worms 72 h post-L1 stage on *E. coli* OP50, WT-STM, *E. coli* OP50 supplemented with vitamin B12, Δ*cobB*, Δ*cobC*, Δ*cobD*, Δ*cobS*, Δ*cbiB*, vitamin B12 supplemented with Δ*cobB*, Δ*cobC*, Δ*cobD*, Δ*cobS*, and Δ*cbiB*, respectively. The *cbiB* mutant reduces the oocyte production, with the reversal observed in vitamin B12 supplementation. Panel **C** represents the quantification of embryos in N2 worms 56 h post-L1 stage, fed on *E. coli* OP50, WT-STM, *E. coli* OP50 supplemented with methionine, Δ*metC*, Δ*metE*, Δ*metH*, vitamin B12 supplemented with Δ*metC*, Δ*metE*, and Δ*metH*, respectively. *metE* and *metH* mutants decreased embryo and (**D**) oocyte production, with the reversal observed in methionine supplementation, respectively. An ordinary one-way ANOVA and a post hoc Tukey HSD test were performed. ****P < 0.0001; ***P < 0.001; **P < 0.01; *P < 0.05*.*

**Fig 4:**
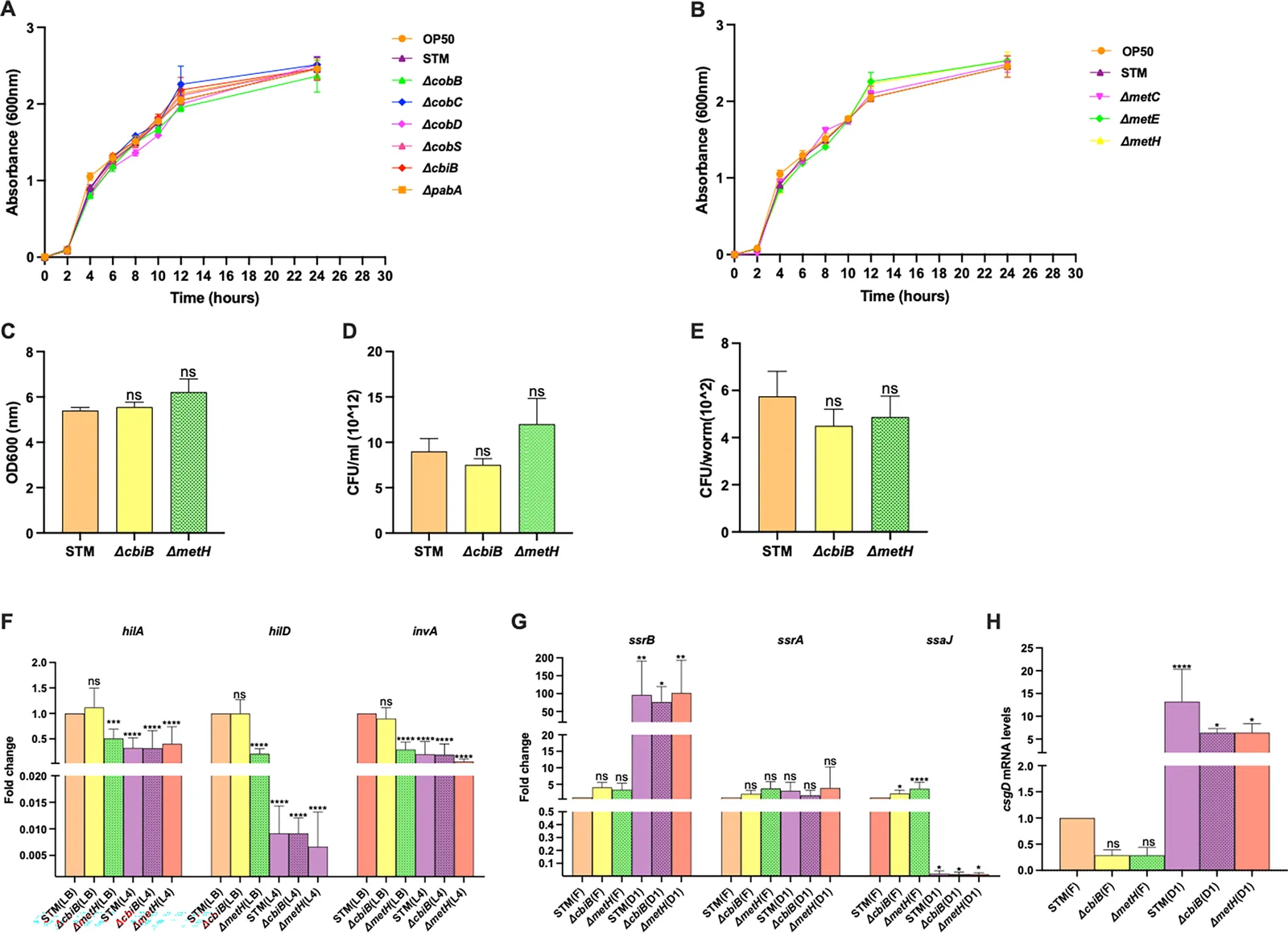
Characterization of *Salmonella* Typhimurium cobalamin and methionine biosynthetic mutants during *in vitro* growth and *C. elegans* colonization**.** (**A and B**) *In vitro* bacterial growth kinetics of WT-STM, *E. coli* OP50, and various target biosynthetic deletion mutants over a 24-h period. Absorbance OD600 was measured at regular intervals. (**A**) Growth curves comparing cobalamin synthesis pathway mutants, Δ*cobB*, Δ*cobC*, Δ*cobD*, Δ*cobS*, Δ*cbiB*, and folate precursor mutant Δ*pabA* against WT-STM. (**B**) Growth curves evaluating methionine pathway mutants Δ*metC*, Δ*metE*, and Δ*metH* with WT-STM. (**C–E**) *In vitro* density and *C. elegans* intestinal colonization efficiency. (**C**) Final culture OD600 and (**D**) colony-forming units per milliliter (CFU/mL) of stationary-phase cultures for WT-STM, Δ*cbiB*, and Δ*metH*. (**E**) Total bacterial colonization load extracted from D1 *C. elegans* intestines (CFU/worm) following feeding on WT-STM, Δ*cbiB*, and Δ*metH* strains. (**F–H**) Relative transcription dynamics of SPI and biofilm regulatory genes determined via qRT-PCR. (**F**) Fold change expression profiles of canonical SPI-1 invasion and virulence genes (*hilA*, *hilD*, and *invA*) in bacteria isolated from L4-stage *C. elegans* (L4), compared to the LB broth subcultured bacteria for 3 h, mimicking SPI-1 activation, and (**G**) relative expression of SPI-2 intracellular survival genes (*ssrB*, *ssrA*, and *ssaJ*) in bacteria isolated from day 1 adult *C. elegans* compared to the F-media, subcultured for 6 h, mimicking SPI-2 activation. (**H**) Relative fold change of *csgD* mRNA transcripts, regulating curli fimbriae and biofilm architecture, in bacteria isolated from day 1 adults compared to the F-media, subcultured for 6 h, mimicking SPI-2 activation. An ordinary one-way ANOVA and a post hoc Tukey HSD test were performed. ****P < 0.0001; ***P < 0.001; **P < 0.01; *P < 0.05.

Having confirmed that the developmental acceleration phenotype depends structurally on bacterial *cbiB* and *metH*, and is entirely uncoupled from differences in bacterial abundance or gut colonization capacity, we next asked whether *Salmonella* actively modulates these pathways within the host environment. We monitored the transcriptional activity of wild-type *Salmonella* harvested directly from the *C. elegans* intestine at the day 1 adult stage [STM(D1)] and compared it with that of control bacteria cultured in standard F-media [STM(F)]. Our *in vivo* expression revealed a striking transcriptional upregulation of the vitamin B12 axis inside the *C. elegans* intestine (Fig. 5A and B). Specifically, the core cobalamin biosynthesis gene *cbiB* exhibited increased transcript levels in the STM(D1) group relative to the F-media control. Similarly, the expression of the cobalamin-dependent methionine synthase *metH* was significantly upregulated. These findings demonstrate that the *C. elegans* gut microenvironment serves as a physiological trigger, stimulating *Salmonella* to actively upregulate endogenous vitamin B12 synthesis and utilization pathways. Thus, these findings together suggest that *S.* Typhimurium accelerates development in *C. elegans* predominantly through dietary vitamin B12, rather than through mechanisms associated with infection or virulence.

**Fig 5:**
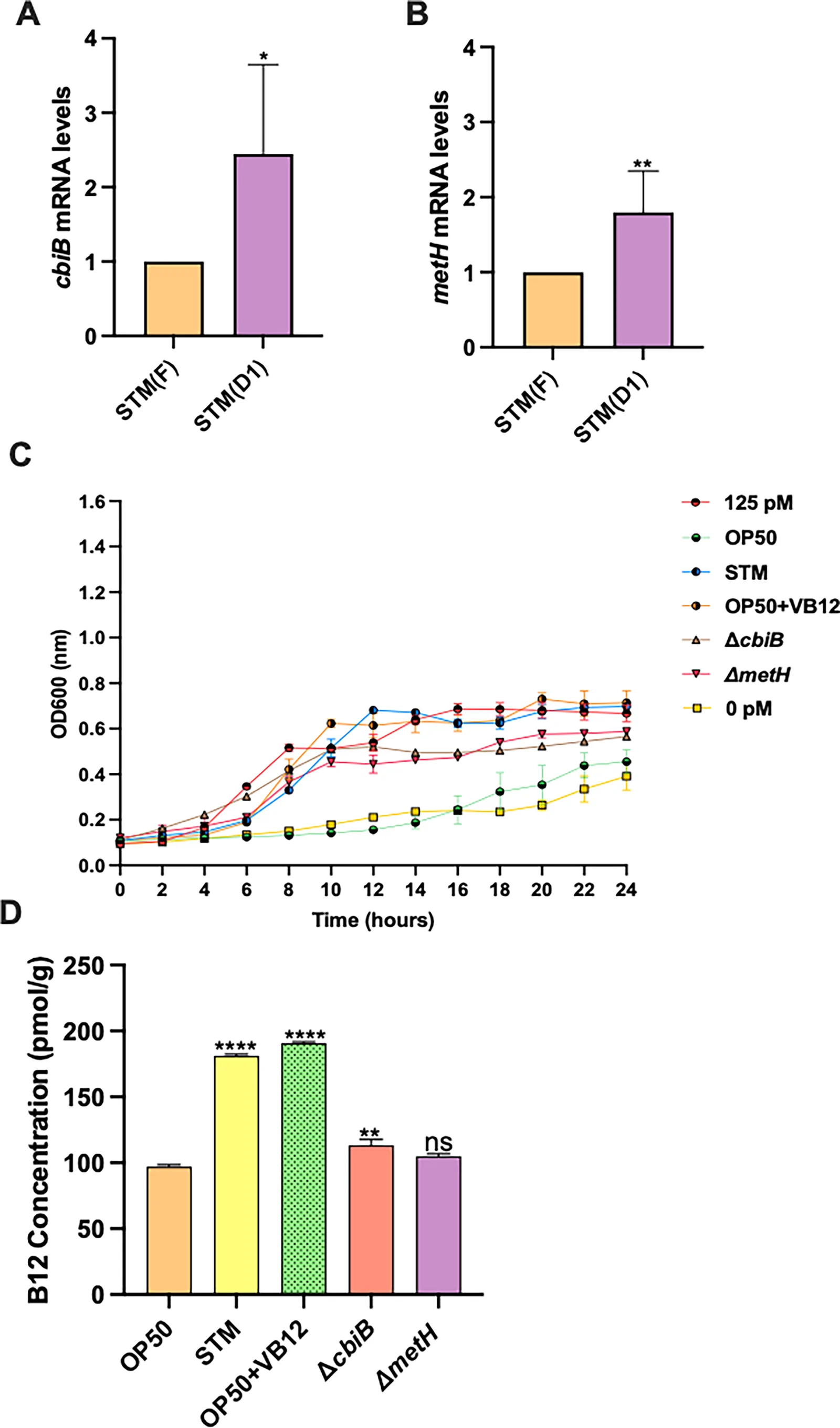
Transcriptional induction of bacterial vitamin B12 metabolic machinery and absolute quantification of internal cobalamin concentrations. (**A and B**) Quantitative qRT-PCR analysis of key cobalamin and methionine pathway genes in *S. Typhimurium* isolated from D1 adult *C. elegans*. (**A**) Relative *cbiB* mRNA levels and (**B**) *metH* mRNA levels in WT-STM isolated from day 1 adulthood compared to the F-media, subcultured for 6 h, mimicking SPI-2 activation. Statistical significance was determined using an unpaired Student’s *t*-test; **P < 0.01; *P < 0.05. (**C and D**) Bioassay-driven quantification of absolute internal vitamin B12 levels in different bacterial conditions. (**C**) Bacterial growth kinetics OD600 of the Δ*metE* monitored over a 24-h window under optimized nutrient-limiting assay conditions, 125 pM vitamin B12 reference standard paired with a 10^3^-fold dilution of B12 isolated from different strains infected worm tissue lysate. Vitamin B12 reference standards of 125 and 0 pM establish the dynamic tracking range. (**D**) Absolute internal vitamin B12 concentrations pmol/g of worm tissue derived from the calculated area under the curve (AUC) metrics from the kinetic growth profiles across *E. coli* OP50, WT-STM, cobalamin-supplemented *E. coli* OP50 (OP50 + VB12), and mutant backgrounds (Δ*cbiB* and Δ*metH*). An ordinary one-way ANOVA and a post hoc Tukey HSD test were performed. ****P < 0.0001; ***P < 0.001; **P < 0.01; *P < 0.05.

To directly quantify the absolute abundance of intracellular cobalamin delivered to *C. elegans* by WT-STM, Δ*cbiB*, and Δ*metH* diets, we used a highly sensitive microbiological bioassay based on the B12/methionine-auxotrophic *Salmonella* Δ*metE* (63, 64). To validate and optimize the dynamic range of the *Salmonella* Δ*metE* bioassay for *C. elegans* tissue extracts, we evaluated four distinct vitamin B12 standards: 1,000, 500, 250, and 125 pM alongside corresponding serial dilutions of the biological extracts 10^0^, 10^−1^, 10^−2^, and 10^−3^-fold, respectively. However, higher concentrations became oversaturated with cobalamin, exceeding the threshold required for optimal growth kinetics (Fig. S2A through C). To bypass the compounding effects of transient growth lag phases, total bacterial proliferation in response to worm lysates was continuously integrated over 24 h for the 125 pM vitamin B12 standard and 10^−3^-fold serial dilutions *of C. elegans* extracts, using area under the curve (AUC) calculations (Fig. 5C and D). Our quantitative analyses revealed a profound accumulation of endogenous vitamin B12 in nematodes maintained on a WT-STM diet (Fig. 5D). Worms maintained on *E. coli* OP50 exhibited lower vitamin B12 levels. In contrast, those fed on WT-STM showed a highly significant increase in their internal vitamin B12 content, which was similar to that of OP50 supplemented with exogenous vitamin B12. The vitamin B12 content was reduced in *C. elegans* fed with Δ*cbiB* and Δ*metH* strains, but was comparable to *that of E. coli* OP50 (Fig. 5D). Taken together, these data clearly demonstrate that WT-STM increases vitamin B12 levels within *C. elegans*, thereby regulating its reproduction and development.

### *Salmonella* accelerates development through vitamin B12-dependent metabolism in *C. elegans*

The *C. elegans* one-carbon metabolism pathway, primarily involving folate and methionine cycles, is essential for nucleotide synthesis, methylation reactions (65), DNA repair, and gene expression regulation (66). While direct interactions between *Salmonella’s* one-carbon metabolism and the host’s metabolic pathways have not been extensively studied, it’s plausible that the pathogen’s metabolic activities could influence these host pathways. Thus, to further investigate whether *Salmonella’s* vitamin B12-dependent one-carbon metabolism influences host development, we exposed the *metr-1*, *sams-1*, and *mmcm-1* mutant strains of the *C. elegans* to the above exposure conditions and assessed embryo and oocyte production. In *metr-1* mutants, the vitamin B12-dependent methionine synthesis pathway is impaired, and vitamin B12 supplementation did not rescue the defects (19, 67). Thus, the mutant continued to exhibit methionine-deficiency phenotypes, including developmental delays and growth defects (20, 65). Methionine is an essential amino acid required to make SAM, a key methyl donor in many biochemical reactions (68, 69). *E. coli* OP50 supplemented with exogenous vitamin B12 showed growth defects. However, *E. coli* OP50 supplemented with exogenous methionine restores the defective pathway in *metr-1* mutants (Fig. 6B and F), possibly by restoring the methionine and SAM pools and rescuing both growth and reproductive defects in the worm. Δ*cbiB* and Δ*metH*, which are cobalamin and methionine biosynthesis mutants, respectively, caused developmental delays with embryo production comparable to that of *E. coli* OP50 and a defect in oocyte production in *metr-1* worms (Fig. 6B and F). This suggests that methionine availability from the bacterial diet is critical for host rescue. *C. elegans* utilizes the bacterial vitamin B12 from *Salmonella* to enhance its own methionine synthesis through a vitamin B12-dependent methionine synthase pathway, encoded by the *metr-1* gene, which is critical for the worm’s development and reproductive success. However, WT-STM infection could not rescue the growth and developmental defects in *metr-1* animals, indicating that *metr-1* cannot utilize bacterial vitamin B12 to synthesize methionine. Exogenous methionine supplementation in *Salmonella* rescued the *metr-1* developmental delay, resulting in a rapid developmental phenotype and increased oocyte production (Fig. 6C and G). This indicates that the *metr-1* worms cannot metabolize vitamin B12 from the bacterial source to synthesize methionine from homocysteine.

**Fig 6:**
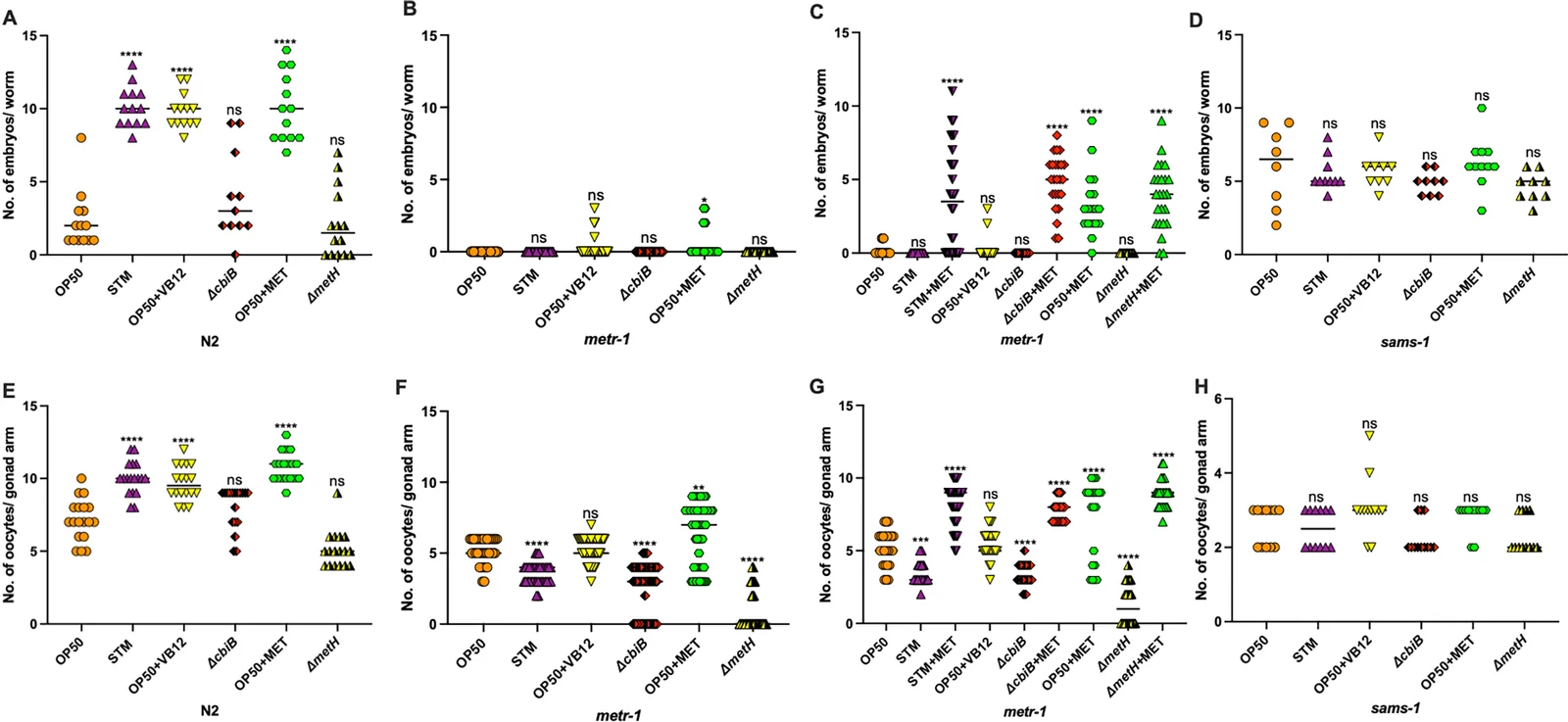
*Salmonella* Typhimurium accelerates development through vitamin B12-dependent metabolism in *C. elegans.* It represents the quantification of embryos in (**A**) N2, (**B**) *metr-1*, and (**D**) *sams-1* on *E. coli* OP50, WT-STM, *E. coli* OP50 supplemented with vitamin B12 and methionine, respectively, Δ*cbiB*, and Δ*metH*. Panel **C** represents the quantification of embryos in *metr-1* on *E. coli* OP50, WT-STM, *E. coli* OP50 supplemented with vitamin B12 and methionine, respectively, Δ*cbiB*, and Δ*metH*, methionine supplementation with WT-STM, Δ*cbiB*, and Δ*metH*, respectively. Panels **E**, **F**, and **H** represent the quantification of oocyte production in N2, *metr-1*, and *sams-1* animals under the indicated bacterial conditions, as mentioned earlier. Panel **G** represents the quantification of oocytes in *metr-1* on *E. coli* OP50, WT-STM, *E. coli* OP50 supplemented with vitamin B12 and methionine, respectively, Δ*cbiB*, and Δ*metH*, methionine supplementation with WT-STM, Δ*cbiB*, and Δ*metH*, respectively. An ordinary one-way ANOVA and a post hoc Tukey HSD test were performed. ****P < 0.0001; ***P < 0.001; **P < 0.01; *P < 0.05.

The *sams-1* mutant displayed growth defects and developmental anomalies (21, 70). Hence, we quantified embryo production at 72 h post-L1 and oocyte number at 96 h post-L1 stage. Similarly, we found reduced embryo and oocyte production when embryos and oocytes were exposed to different bacterial diets, including *E. coli* OP50, WT-STM, and *E. coli* OP50 supplemented with vitamin B12 or methionine, resulting in growth defects (Fig. 6D and H). The *mmcm-1* mutant, defective in the vitamin B12-dependent propionate breakdown pathway, shows no growth defects across these various bacterial diets (Fig. S3A and B). The data suggest that *S*. Typhimurium, a facultative anaerobe, synthesizes vitamin B12 in the gut of *C. elegans*. The *C. elegans* utilizes this vitamin to produce methionine, a precursor of SAM. This process is essential for the rapid development and reproductive maturity of *C. elegans*, and *Salmonella’s* metabolic pathway enhances these processes.

### *Salmonella* enhances vitellogenin activity and mitigates oxidative stress, restoring vitellogenesis and oocyte development

Vitellogenins are yolk proteins synthesized in the adult intestine that supply nutrients to developing oocytes to support embryo development (71, 72). The *C. elegans* fed with WT-STM produced more oocytes (Fig. 2B). As yolk proteins are major components of oocytes, we further examined vitellogenin levels in worms fed on *Salmonella.* We assessed vitellogenin gene 2 (*vit-2*) expression using a *vit-2::GFP* reporter strain. We observed increased expression of VIT-2::GFP in both oocytes (Fig. 7A and B) and embryos (Fig. 7C and D) when *C. elegans* were fed on WT-STM or *E. coli* OP50 supplemented with vitamin B12 or methionine, respectively. In contrast, a comparable VIT-2::GFP expression was observed in *C. elegans* fed on Δ*cbiB* and *ΔmetH* and *E. coli* OP50. Δ*cbiB* and Δ*metH* supplemented with methionine increased the VIT-2::GFP expression in both oocytes (Fig. 7A and B) and embryos (Fig. 7C and D). This suggests that a vitamin B12-rich diet of WT-STM enhances vitellogenesis and developmental growth by modulating methionine metabolism. Methionine supplementation with *E. coli* OP50, *Salmonella*, Δ*cbiB*, and Δ*metH* effectively rescues developmental defects in *metr-1* mutants of *C. elegans* (Fig. 6B, C, F, and G). The *rme-2* gene is the primary yolk receptor essential for importing yolk proteins into oocytes via receptor-mediated endocytosis (73). Additionally, we analyzed mRNA levels of the *vit-2*, *vit-5*, and *rme-2* genes in *metr-1* mutants and found them upregulated (Fig. 7E and F). Moreover, methionine supplementation not only mitigates developmental defects but also significantly enhances the expression of germline signaling transcripts, such as *glp-1* and *mpk-1* in N2 and *metr-1* mutants, respectively (Fig. 7G and H), thereby linking one-carbon metabolism to oocyte proliferation pathways. DA1877 is the canonical high-B12 diet for *C. elegans* and recapitulates B12-dependent effects on development, the methionine/SAM cycle, and VIT-2 expression. The DA-fed control experiments, for embryo and oocyte quantification in N2 and *metr-1* worms, and VIT2::GFP quantification, show the expected B12-dependent phenotypes, confirming that the phenotype observed is due to a high level of vitamin B12 supplied by *Salmonella* (Fig. S4A through F), and Δ*cbiB* and Δ*metH* mutant strains of B12 synthesis lost the phenotypes. Methionine supplementation in DA-fed *metr-1* worms rescued the phenotype. These data clearly indicate the B12-mediated regulation of the *C. elegans* reproductive development.

**Fig 7:**
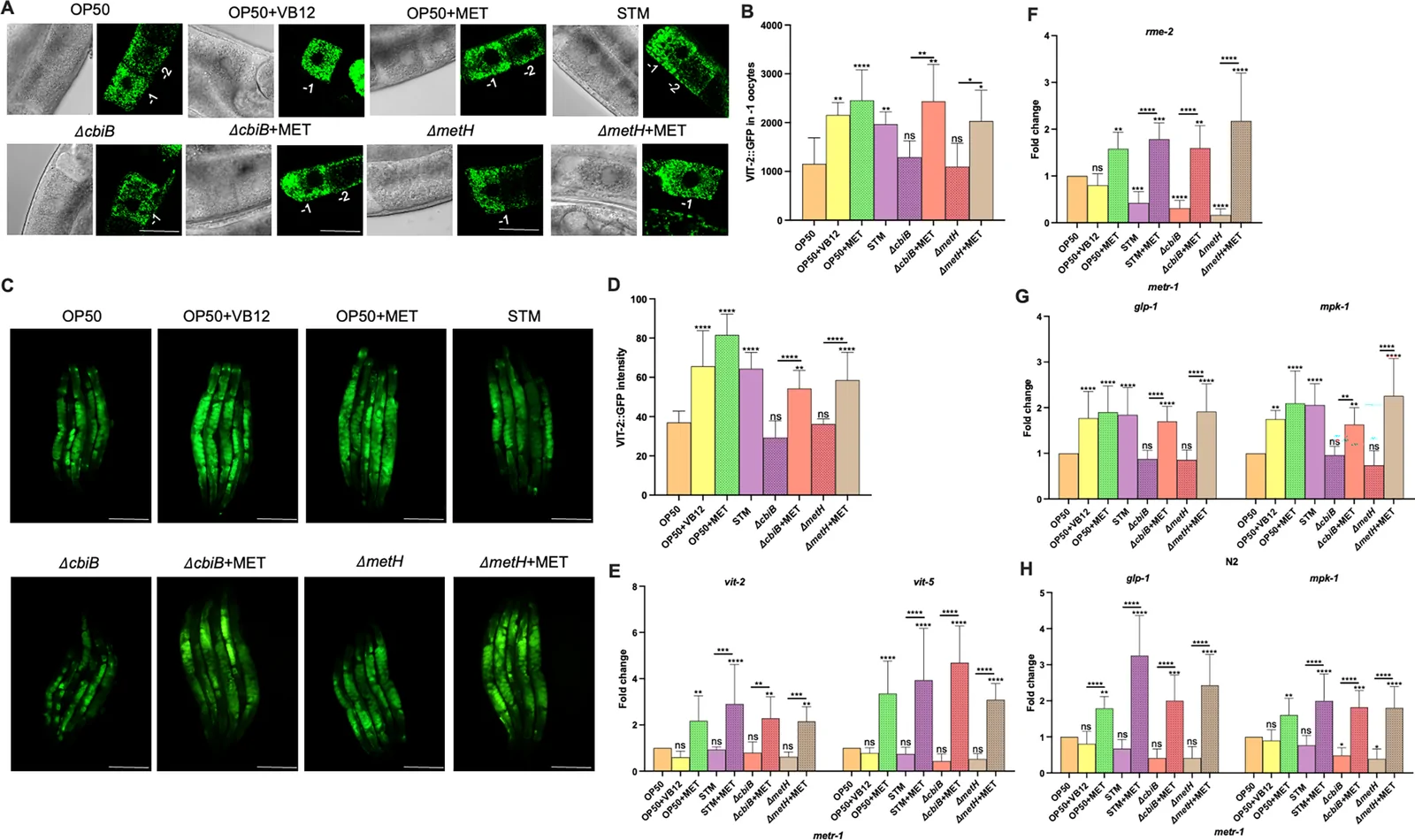
*Salmonella* Typhimurium increases vitellogenin activity, restoring vitellogenesis and oocyte development. (**A**) Representative confocal microscopy images of the vitellogenin-2 expression, using *vit-2::gfp* transgenic worms in the primary oocytes of day 1 adults fed on *E. coli* OP50, wild-type *Salmonella*, Δ*cbiB*, Δ*metH*, *E. coli* OP50 supplemented with either vitamin B12 or methionine, respectively. (**B**) Quantification of vitellogenin-2 expression in the primary oocytes of day-1 adults under the indicated bacterial conditions. (**C**) The representative images and (**D**) quantification of vitellogenin-2 expression in the embryos of day 1 adults under the indicated bacterial conditions. (**E**) mRNA expression levels of vitellogenin genes, *vit-2* and *vit-5*, (**F**) yolk receptor *rme-2* gene. (**G and H**) The germline proliferation gene, *glp-1*, and ERK-encoding gene, *mpk-1*, show upregulation in N2 and *metr-1* mutants, when exposed to methionine supplementation with *E. coli* OP50, *Salmonella*, Δ*cbiB*, and Δ*metH*, respectively. An ordinary one-way ANOVA and a post hoc Tukey HSD test were performed. ****P < 0.0001; ***P < 0.001; **P < 0.01; *P < 0.05*.* Scale bar = 100 µm.

Vitamin B12 has also been shown to exhibit antioxidant activity, which helps mitigate oxidative stress (74, 75). We observed increased VIT-2 expression and oocyte production in *C. elegans* fed on *Salmonella* driven by vitamin B12-dependent methionine metabolism. Reactive oxygen species (ROS) are cleared by antioxidant enzymes, such as GST-4, a glutathione S-transferase regulated by *skn-1* during oxidative stress response (76–78). Therefore, we examined GST-4 levels using the *gst-4::GFP* reporter strain, which was fed on WT-STM or *E. coli* OP50 supplemented with vitamin B12 or methionine, respectively. Consistent with the role of vitamin B12, we found reduced GST-4 expression in WT-STM and *E. coli* OP50 supplemented with vitamin B12 or methionine, respectively, while *E. coli* OP50, Δ*cbiB*, and Δ*metH* fed *C. elegans* displayed increased GST-4 activation (Fig. 8A and B). ROS levels were measured by the intensity of dichlorofluorescein (DCF), which was significantly higher in worms fed diets of *E. coli* OP50, Δ*cbiB*, and Δ*metH* compared to those on the vitamin B12-rich diet of WT-STM, *E. coli* OP50 supplemented with B12, and methionine, respectively (Fig. 8C). These findings indicate that the vitamin B12-rich diet enhanced vitellogenin activity to help reduce oxidative stress in *C. elegans* fed on WT-STM. To confirm that the effect depends on methionine metabolism, we assessed ROS levels in the *metr-1* mutant, which cannot acquire dietary vitamin B12, a cofactor required for methionine synthesis, leading to decreased reproductive maturity (Fig. 6B, C, F, and G). This suggests reduced vitellogenesis and increased oxidative stress. ROS levels in *metr-1* worms on vitamin B12-rich diets were comparable to *E. coli* OP50, Δ*cbiB*, and Δ*metH* (Fig. 8D). A vitamin B12-rich diet enhances vitellogenin activity and reduces oxidative stress, likely through shared effects on methionine metabolism rather than direct antioxidant effects. While elevated vitellogenin is associated with lower oxidative stress, evidence from the *metr-1* mutant indicates that it may not directly reduce ROS. Instead, vitellogenin seems to serve as an indicator of metabolic health, influenced by methionine and the SAM cycle. Interestingly, *Salmonella* is reported to induce oxidative stress in *C. elegans* when L4 nematodes are acutely exposed for 24 h (79). However, in our experimental paradigm, feeding differed, as we exposed L1 larvae to *Salmonella* until they reached adulthood. This condition may result in reduced ROS because *C. elegans* consumes *Salmonella* as its diet, leading to increased expression of VIT-2 and indicating enhanced antioxidant activity, which consequently reduces ROS. We observed that the vitellogenin levels in the nematodes decreased after 24 h of exposure to *Salmonella* Typhimurium. In contrast, VIT-2 expression increased upon supplementation with *E. coli* OP50, vitamin B12, and methionine (Fig. S5A and B). This finding was further validated using the *gst::4-GFP* reporter strain, which showed no significant change in GST-4 expression compared to *E. coli* OP50 but exhibited reduced expression during *E. coli* OP50 supplementation with vitamin B12 and methionine, respectively (Fig. S5C and D). The methionine cycle also contributes to cysteine synthesis, a precursor for glutathione (80, 81), an important antioxidant that lowers oxidative stress, which otherwise suppresses VIT-2 expression (82) and ultimately supports oocyte development. Rather than vitellogenin acting directly as an antioxidant, methionine metabolism feeds into cysteine and glutathione synthesis, thereby lowering oxidative stress and indirectly supporting vitellogenin expression (34). Methionine metabolism is a key regulator of lifespan in *C. elegans*. Methionine restriction extends lifespan, while effective cycling is vital for dietary and metabolic interventions that promote longevity (83). Its links to reproduction, stress response, and epigenetics make methionine an important factor in aging in nematodes (82, 84). We observed that WT-STM exposure caused early mortality in worms, while *E. coli* OP50 supplemented with methionine and Δ*cbiB* exposed worms showed no significant survival difference compared to *E. coli* OP50. However, *E. coli* OP50 supplemented with vitamin B12 and Δ*metH* led to longer survival than the other bacterial diets (Fig. S5E). Methionine metabolism plays a crucial role in the growth and virulence of *S.* Typhimurium; mutants deficient in these pathways are less virulent (13). This suggests the role of metabolic and virulence competence in *S.* Typhimurium, highlighting the importance of these metabolites to both the pathogen and the host (85, 86).

**Fig 8:**
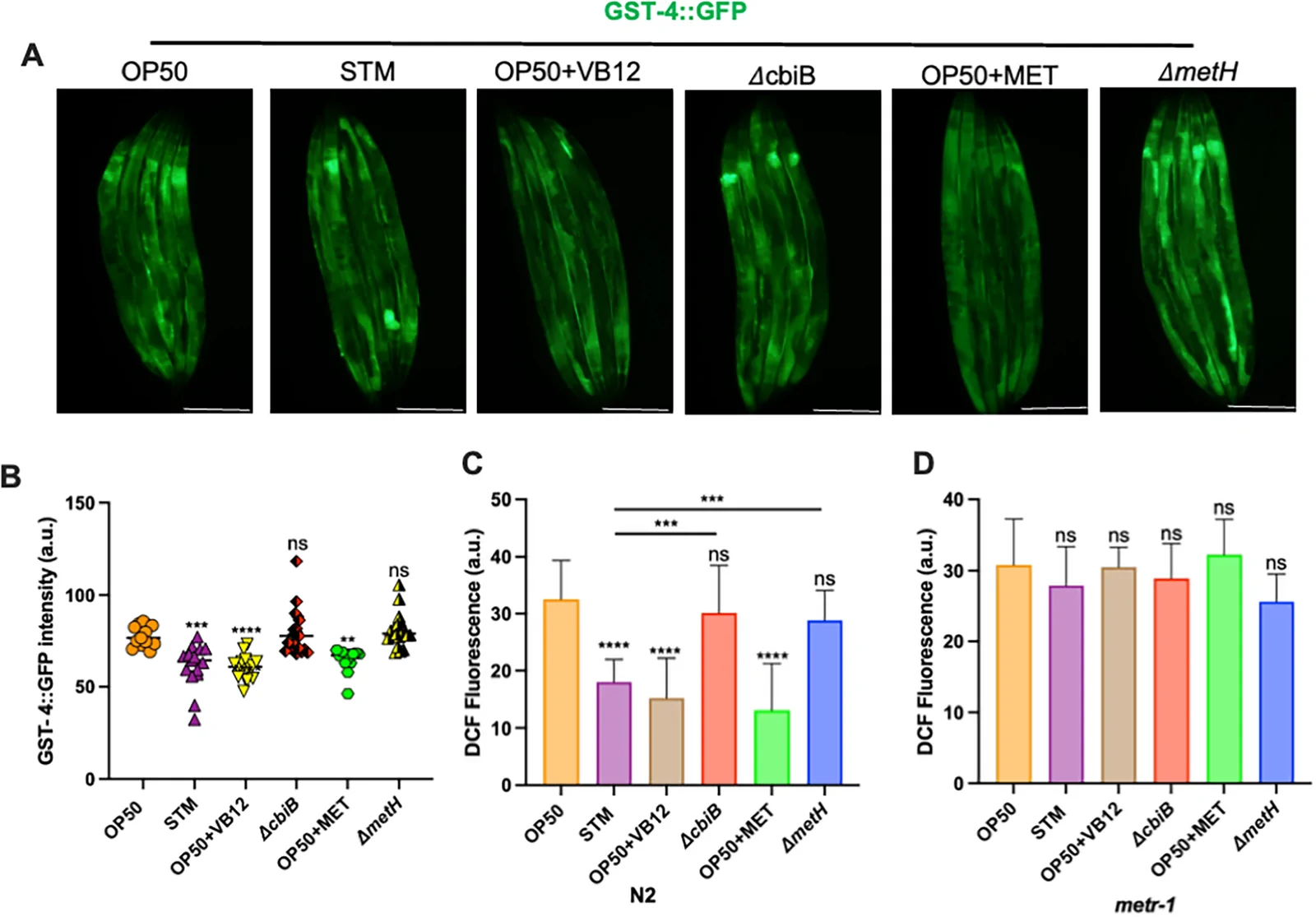
*Salmonella* enhances methionine metabolism, promoting vitellogenin expression and reducing oxidative stress. (**A**) Representative fluorescence images and (**B**) quantification of GST-4::GFP expression in day 1 adult worms under the indicated bacterial conditions. Quantification of fluorescence levels of 2′,7′-dichlorofluorescein (DCF) in (**C**) N2 and (**D**) *metr-1* animals under the indicated bacterial conditions. An ordinary one-way ANOVA and a post hoc Tukey HSD test were performed. ****P < 0.0001; ***P < 0.001; **P < 0.01; *P < 0.05*.* Scale bar = 100 µm.

### *Salmonella* exposure decreases mitochondrial activity by reducing its biogenesis

Vitamin B12 decreases the function of HSP-6, a key component of the mitochondrial unfolded protein response in *C. elegans*, and regulates mitochondrial stress response (87). We aimed to assess mitochondrial health by monitoring the expression of the mitochondrial stress reporter transgene, *hsp-6::GFP*, and measuring mitochondrial genomic DNA copy number. The *hsp-6::GFP* transgenic worms were exposed to the bacterial conditions mentioned above. Under WT-STM, *E. coli* OP50 supplemented with vitamin B12 or methionine, the relative mean fluorescence intensity of HSP-6::GFP was significantly decreased. In contrast, the GFP levels were comparable in *E. coli* OP50, Δ*cbiB*, and Δ*metH* fed to *C. elegans*, suggesting that vitamin B12-mediated methionine metabolism can maintain nematodes’ proteotoxic stress response (Fig. 9A and B). The mtDNA copy number is a marker of mitochondrial biogenesis, as mtDNA replication is integral to this process (88). To examine the mitochondrial biogenesis rate, we analyzed the mtDNA copy number using the mitochondrial ATP synthase gene (*atp-6*), which is crucial for ATP synthesis. Normalization was performed with the housekeeping nuclear gene *ama-1*. The mtDNA copy number is decreased in WT-STM, *E. coli* OP50 supplemented with vitamin B12 or methionine. At the same time, Δ*cbiB-* and Δ*metH*-fed worms showed levels comparable to *E. coli* OP50 (Fig. 9C). Thus, the decreased mitochondrial gDNA and HSP-6::GFP expression in high vitamin B12 diets suggests that increased vitamin B12 may inhibit mitochondrial biogenesis and stress response activation, reflecting improved mitochondrial health and proteostasis.

**Fig 9:**
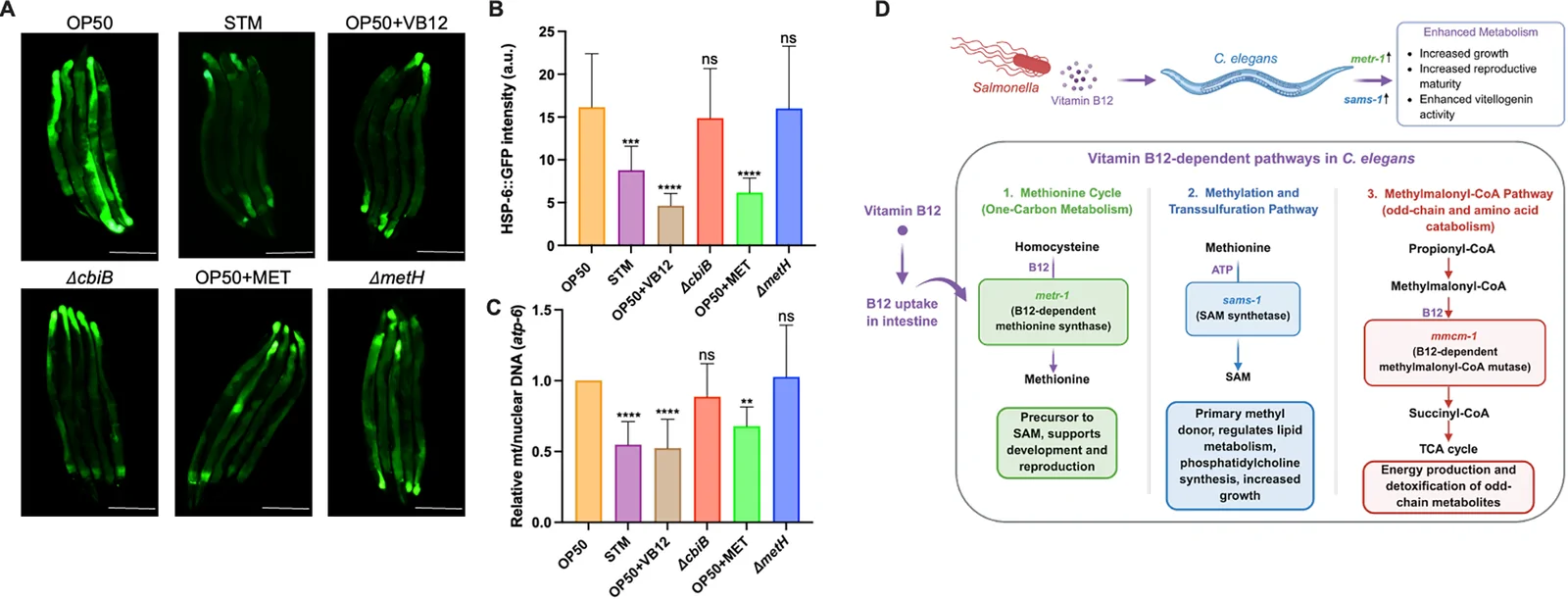
*Salmonella* Typhimurium decreases mitochondrial activity. (**A**) Representative images and (**B**) quantification of GFP levels of *hsp-6::gfp* worms on *E. coli* OP50, wild-type *Salmonella*, Δ*cbiB*, Δ*metH*, *E. coli* OP50 supplemented with either vitamin B12 or methionine, respectively. HSP-6::GFP expression is reduced in worms grown on WT-STM and on *E. coli* OP50 supplemented with vitamin B12 and methionine, respectively. Panel **C** represents the quantification of total mtDNA copy number from day 1 adult N2 worms on the indicated bacterial conditions by qPCR. WT-STM, *E. coli* OP50 supplemented with vitamin B12 and methionine, respectively, reduces mitochondrial DNA content, while animals fed with *cbiB* and *metH Salmonella* mutants show increased levels comparable to *E. coli* OP50. (**D**) *Salmonella* infection induces a diet-dependent metabolic shift in *C. elegans* via vitamin B12 pathways. Dietary vitamin B12 derived from *Salmonella* is absorbed in the *C. elegans* intestine, driving three major parallel pathways that promote an enhanced metabolic phenotype, including increased growth, accelerated reproductive maturity, and elevated vitellogenin activity. An ordinary one-way ANOVA and a post hoc Tukey HSD test were performed. ****P < 0.0001; ***P < 0.001; **P < 0.01; *P < 0.05*.* Scale bar = 100 µm.

## DISCUSSION

Our previous study from the lab showed that *Salmonella* infection shortened the lifespan and accelerated developmental progression in *C. elegans* (16). This study demonstrates that *Salmonella* Typhimurium accelerates development and reproductive maturity in *C. elegans* through a vitamin B12-dependent mechanism, rather than via virulence factors (Fig. 1 to 4). The relationship between infection and reproduction involves a complex trade-off in which organisms balance immune defense and reproductive effort (89). Understanding immune-reproductive interactions is crucial for developing effective prevention and treatment strategies within the host-pathogen dynamics. *C. elegans* has been used as a model to understand the immunity-reproduction trade-offs and pathogen-specific reproductive strategies (90, 91). Exposure to pathogenic *Pseudomonas aeruginosa* reduces brood size and causes germline shrinkage, whereas exposure to the *gacA* mutant, which is defective in virulence, reverses the germline defect (92). The *Salmonella fepB* enhanced dauer formation and stress resistance in *C. elegans* during infection (40). We also exposed the worms to various avirulent *Salmonella* strains, including SPI-I and SPI-II, as well as non-SPI mutant strains, as listed in Table 1. We observed that these mutants still increased the developmental rate and reproductive maturity, as observed by increased embryo and oocyte production (Fig. 1A and B). Our data contradict the “stress-induced terminal investment” hypothesis, which suggests that a host accelerates reproduction to maximize fitness before succumbing to a pathogen (93–95). During the critical L1–L4 reproductive acceleration window, canonical SPI-1 genes *hilA*, *hilD*, and *invA* remain strictly uninduced (Fig. 4F). At the day 1 adult stage, the SPI-2 system shows a distinct transcriptional uncoupling: the sensor kinase *ssrA* stays basal, while the response regulator *ssrB* is highly upregulated. Crucially, the structural secretion component *ssaJ* is downregulated (Fig. 4G), whereas the master biofilm regulator *csgD* is upregulated (Fig. 4H). Rather than executing cytotoxicity, this molecular signature reflects *ssrB*’s ancestral role in optimizing non-toxic luminal survival and biofilm accumulation (96–98). Furthermore, while Δ*cbiB* and Δ*metH* mutants colonize the intestine normally, they fail to accelerate development, proving the phenotype is biochemical rather than virulent (Fig. 4E). The significant *in vivo* upregulation of *cbiB* and *metH* mRNA levels at the day 1 adult stage demonstrates that the host intestinal environment actively triggers *Salmonella* to induce its endogenous vitamin B12 biosynthetic and metabolic machinery (Fig. 5A and B). Thus, this developmental response is strictly driven by internal vitamin B12 metabolic machinery rather than pathogen-induced host stress. Different bacterial diets are a major determinant of *C. elegans* physiology and life-history traits. For instance, a *Comamonas* DA1877 diet accelerates development but reduces fecundity and shortens lifespan compared to the *E. coli* OP50 diet, and these effects are attributed to its nutritional composition rather than to pathogenicity (60). Vitamin B12 is vital for methionine synthesis and methylmalonyl-CoA metabolism, making it indispensable for cellular energy production, DNA synthesis, and nervous system maintenance (99, 100). The microbiota’s role is essential to host metabolism, and it provides a framework for understanding the complex interactions among diet, microbiota, metabolism, and disease (20).

*Salmonella hilA* mutant and vitamin B12 supplementation showed comparable progeny and oocyte numbers to those of WT-STM (Fig. 2A through C), indicating that bacterial metabolism, rather than virulence factors, is involved in regulating reproductive development. *Salmonella* exhibits a vitamin B12-rich diet compared to *E. coli* OP50 (Fig. 2D and 5D), which contributes to its rapid development in *C. elegans.* Our findings highlight that organisms traditionally classified as pathogens may also benefit their hosts. This duality complicates our understanding of host-microbe interactions and highlights the need for a more detailed approach to microbial ecology. We found that *Salmonella* mutants deficient in cobalamin (*cbiB*) and methionine (*metH*) biosynthesis pathways reduced the developmental acceleration observed in *C. elegans* when fed a vitamin B12-rich diet (Fig. 3A through D), indicating a crucial role for these pathways in development. Dietary regulation of cellular metabolism requires adaptations related to vitamin B12 and one-carbon metabolism. Specifically, the folate cycle influences germline development (101), while metabolites of the methionine cycle enhance RAS/MAPK traits (65). The *metr-1* mutant of the worm, defective in vitamin B12-dependent methionine synthesis, did not show accelerated development when exposed to WT-STM and *E. coli* OP50 supplemented with vitamin B12, suggesting that *C. elegans* utilizes bacterial vitamin B12 to enhance its methionine synthesis (Fig. 6B and F), a process that contributes to its development. In the *metr-1* mutant, the absence of functional methionine synthase disrupts the methionine cycle, preventing the conversion of dietary vitamin B12 into its active forms and resulting in impaired embryo and oocyte production (102). These defects are likely due to impairments in downstream processes, such as SAM production and histone methylation, which are vital for oogenesis (103). Methionine supplementation restored normal embryonic development and oocyte production in the *metr-1* mutant compared to WT-STM (Fig. 6C and G). Vitamin B12 is essential for regulating reproduction in *C. elegans* by enhancing vitellogenin production through the methionine/SAM cycle (31, 87). This process affects gene expression (Fig. 7E through H), improves oocyte quality, and optimizes reproductive timing, allowing organisms to adjust their strategies in response to dietary metabolites and the microbiota (65, 104, 105). Methionine supplementation increased antioxidant defense by elevating superoxide dismutase, catalase, and glutathione peroxidase activity while reducing malondialdehyde levels, thus mitigating oxidative stress during heat exposure (106). *S*. Typhimurium exposure increases vitellogenin production (Fig. 7A through D) and decreases oxidative stress (Fig. 8A through C) in *C. elegans*, likely by improving methionine metabolism. Thus, vitellogenin acts as an antioxidant, protecting organisms from ROS and enhancing resistance to oxidative stress. This protective role has been reported in various species, including nematodes, insects, fish, and vertebrates (33, 107, 108).

Mitochondrial abnormalities in oocytes can be inherited, leading to developmental defects and increasing the risks of metabolic disorders in offspring, underscoring the importance of mitochondrial function for transgenerational health (109, 110). We found that mitochondrial function was altered in *S*. Typhimurium-fed worms, with decreased mitochondrial stress responses and biogenesis (Fig. 9A and B). These results highlight the complex metabolic interactions between bacteria and their host (111). *Salmonella*, often seen as a pathogen, provides nutritional benefits due to its high vitamin B12 content, which enhances methionine metabolism in the host and promotes faster development and reproduction. This challenges the view of microbes as merely pathogens, suggesting that *C. elegans* may benefit from *Salmonella* during interaction. The study highlights the implications of vitamin B12 deficiency for other organisms, including humans, and demonstrates that altered mitochondrial function in these worms results in increased mitochondrial stress responses (112, 113). Increased vitellogenin production and reduced oxidative stress underscore the positive effects of bacterial metabolites on reproductive health. Early oocytes exhibit a mitochondrial adaptation that maintains low ROS levels during metabolism, crucial for oocyte quality and developmental potential (114, 115). Vitamin-rich diets led to a reduced number of mitochondria, with a stable membrane potential (116, 117). It also significantly decreased ROS production. These findings suggest that a dietary deficiency in vitamin B12 has a severe impact on mitochondrial health and function. ROS may play a role in regulating mtDNA copy number (118). mtDNA and nuclear genes encode proteins for oxidative phosphorylation. mtDNA is prone to oxidative damage from ROS, leading to mutations linked to aging and mitochondrial disorders (119). Our findings show that exposure to a vitamin B12-rich diet decreases ROS levels (Fig. 8A through C), leading to the inactivation of mitoUPR in worms (Fig. 9A and B), thereby maintaining mitochondrial homeostasis (120). The observed phenotype in *C. elegans* fed on WT-STM consists of earlier reproduction and reduced lifespan (Fig. S5E). While vitamin B12 is well-documented to promote larval development, its downstream effects on longevity appear highly conditional. Dietary cobalamin deficiency impairs fertility and reduces lifespan, whereas adequate baseline levels optimize metabolic flux and increase tolerance to environmental stress (23, 121). Consequently, our findings argue against the notion that vitamin B12 acts as a universal determinant of shortened lifespan; rather, it functions as a context-dependent regulator that shapes complex life-history traits in a diet- and environment-specific manner. The observed reductions in baseline mitochondrial biogenesis and stress pathways (Fig. 9 and Fig. S5) suggest a stabilization of homeostasis rather than a failure. By clearing metabolic bottlenecks, vitamin B12 enhances cellular efficiency and reduces reliance on energy-intensive stress-response mechanisms (122, 123). Collectively, the synergistic upregulation of *metr-1* and *sams-1* in response to *Salmonella*-derived nutrient influx shifts the *C. elegans* into an enhanced metabolic state, characterized by accelerated growth, early reproductive maturity, and elevated vitellogenin activity (Fig. 9D), while *mmcm-1* was found to have no role in the developmental acceleration phenotype observed in *C. elegans* fed on WT-STM.

In conclusion, this study reveals a metabolic interaction between *S*. Typhimurium and *C. elegans* mediated by bacterial vitamin B12, highlighting the role of microbial metabolism in influencing nematode development and reproductive maturity. The full molecular mechanisms underlying *Salmonella*’s effects on vitamin B12 are likely more complex and may require in-depth exploration using proteomics, metabolomics, and other omics approaches.

## MATERIALS AND METHODS

### *C. elegans* culture and maintenance

*C. elegans* N2 (Bristol) strain was used as a wild type and maintained at 20°C on standard NGM plates seeded with *Escherichia coli* OP50. Before the experiment, synchronized L1 larvae were harvested from the gravid *C. elegans* using 5% sodium hypochlorite and 5 N NaOH and incubated for 20 h in an M9 buffer (KH_2_PO_4_ 0.3%, Na_2_HPO_4_ 0.6%, NaCl 0.5%, 1 mM MgSO_4_) with gentle shaking at room temperature. The transgenic worm used in this study: OD95: *ltIs37 [pie-1p::mCherry::his-58 + unc-119(+)] IV. ltIs38 [pie-1p::GFP::PH(PLC1delta1) + unc-119(+)]*, VL749: *wwIs24 [acdh-1p::GFP + unc-119(+)]*, CL2166: *dvIs19 [(pAF15)gst-4p::GFP::NLS] III*, SJ4100: *zcIs13 [hsp-6p::GFP + lin-15(+)]*, RB755*: metr-1(ok521) II*, RB1434*: mmcm-1(ok1637) III*, VC2428: *sams-1(ok2946)*, and DH1033: *bIs1 [vit-2::GFP + rol-6(su10060)] X.* All transgenic worms are maintained at 20°C.

### Bacterial strains, media, and growth conditions

The following bacterial strains were used: *Escherichia coli* OP50 and *Salmonella* Typhimurium 14028, which served as the wild-type parent strain. *Escherichia coli* OP50, *Comamonas aquatica* DA1877, and *Salmonella* Typhimurium were cultured in LB broth at 37°C. Δ*hilA*, SPI-1 effector mutants, and Δ*ssaV* SPI-2 mutant, *E. coli* containing pKD4 strain, were grown in LB broth with kanamycin (50 µg/mL) at 37°C, and Δ*phoP* was *grown* in LB broth with chloramphenicol (50 µg/mL) at 37°C*. S*. Typhimurium carrying pKD46 was cultured in LB broth with ampicillin (50 µg/mL) at 30°C. Δ*cobB*, Δ*cobC*, Δ*cobD*, Δ*cobS*, Δ*cbiB*, Δ*pabA*, Δ*metC*, Δ*metE*, and Δ*metH Salmonella* mutants generated in the laboratory, as described below, were used for this study. These mutants were grown in LB broth with kanamycin (50 µg/mL) at 37°C.

### Generation of cobalamin, folate, and methionine biosynthesis mutant strains of *Salmonella* Typhimurium

*S.* Typhimurium mutant strains were generated using a one-step gene inactivation process (124). Briefly, the *Salmonella* carrying the red helper plasmid pKD46 was grown overnight in LB medium with ampicillin at 30°C. This overnight culture was subcultured at a 1:100 ratio in Super Optimal Broth medium supplemented with 1 M MgSO_4_, 1 M MgCl_2_, and 1 M arabinose, and then incubated until the optical density reached 0.4–0.6. Electrocompetent cells were prepared by washing the culture with ice-cold 10% glycerol. The kanamycin cassette from the pKD4 plasmid was amplified using cassette-specific primers mentioned in Table 2 for the genes of interest (*cobB*, *cobC*, *cobD*, *cobS*, *cbiB*, *pabA*, *metC*, *metE*, and *metH*) and purified. The PCR product was transformed into the pKD46-*Salmonella* strain by electroporation, then a pre-warmed Super Optimal Broth with Catabolite Repression (SOC) medium was added and incubated at 30°C overnight. The culture was then plated on LB agar containing kanamycin (50 µg/mL) at 37°C for 12 h, and kanamycin-resistant mutants were confirmed by colony PCR.

**TABLE 2:** Developmental rate of *C. elegans* N2, *metr-1*, *mmcm-1*, and *sams-1* in response to wild-type *Salmonella*, Δ*cbiB*, and Δ*metH* mutants

Strain	Genotype	Metabolic pathway	Developmental rate
N2	Wild type (Bristol)	Reference	Increased on WT-STM, while reduced on Δ*cbiB* and Δ*metH*
RB755	*metr-1*	Vitamin B12-dependent methionine synthesis (20, 60)	Reduced on WT-STM, Δ*cbiB*, and Δ*metH*, while increased with methionine supplementation
VC2428	*sams-1*	S-adenosylmethionine (SAM) biosynthesis (125)	Reduced on WT-STM, Δ*cbiB*, and Δ*metH*, no changes with methionine supplementation
RB1434	*mmcm-1*	Vitamin B12-dependent propionate metabolism (19, 60)	Increased on WT-STM, while reduced on Δ*cbiB* and Δ*metH*; similar phenotype as that of N2

### Exposure of *C. elegans* to *Salmonella* and its mutant strains

Synchronized L1-stage larvae were exposed to wild-type *Salmonella* (WT-STM) and its mutants. The worms were then allowed to grow for 56 h until they reached young adulthood, and embryo production was quantified. Day 1 adulthood was used to quantify oocyte production.

### Exposure of *C. elegans* to *Comamonas aquatica* DA1877

Synchronized L1-stage larvae were exposed to DA1877. The worms were then allowed to grow for 56 h until they reached young adulthood, and embryo production was quantified. Day 1 adulthood was used to quantify oocyte production and VIT-2::GFP expression.

### Brood size analysis

Brood size of *C. elegans* was determined as described previously (126), with slight modifications by placing a single late L4 hermaphrodite in a freshly seeded plate with *E. coli* OP50, WT-STM, Δ*hilA*, and *E. coli* OP50 supplemented with vitamin B12, allowing it to lay eggs for 24 h, and transferring it to a fresh plate afterward. This was repeated every 24 h until the animal stopped laying eggs. Viable progenies (L4 larvae) were counted in each plate after 48 h and scored. Brood size was expressed as the total number of viable progeny per animal. For each bacterial condition, five hermaphrodite worms were transferred to separate plates, each with one worm.

### Analysis of embryos and oocyte numbers

N2, *metr-1*, *mmcm-1*, and *sams-1* worms were exposed to the bacterial conditions mentioned above for embryo number quantification. Embryos were numbered and scored in 56-hour-old young adult animals in N2, *metr-1*, and *mmcm-1*, while at 72 h in *sams-1* worms. The oocyte counts were quantified in day 1 adult hermaphrodites of N2, *metr-1*, and *mmcm-1* at 96 h post-L1 stage in *sams-1* worms. At the indicated time points, *C. elegans* were washed three times in M9 buffer to remove excess bacteria, anesthetized with 25 mM Levamisole in M9 buffer, mounted on 3% agarose pads on glass slides, covered with coverslips, and subjected to a 40× compound microscope. For oocyte quantification using the OD95 strain, the samples were prepared similarly to those of N2 worms and imaged with a 60× objective lens on a Zeiss laser-scanning confocal microscope (LSM 980).

### Vitamin B12 and methionine supplementation

Vitamin B12 (cobalamin) was purchased in its precursor form, cyanocob(III)alamin (CNCbl) (Sigma V2876). Vitamin B12 was dissolved in sterile Milli-Q water at a 1 mM stock concentration and added at a final concentration of 64 nM in the NGM plates seeded with the indicated bacterial conditions. Methionine (50 mg/mL) stock was prepared in sterile Milli-Q water and added to NGM seeded with the indicated bacterial conditions at a final concentration of 10 mM.

### Quantification of ACDH-1::GFP, VIT-2::GFP, GST-4::GFP, and HSP-6::GFP expression

The reporter strains VL749, DH1033, CL2166, and SJ4100 were used to visualize ACDH-1, VIT-2, GST-4, and HSP-6 expression, respectively. They were synchronized as stated above to obtain age-synchronized L1 larvae. Approximately 200 age-synchronized L1 larvae were transferred to each NGM plate seeded with the indicated bacterial conditions and were incubated at 20°C for 72 h. After that, the worms were prepared for fluorescence imaging.

### Fluorescence imaging

Fluorescence imaging was performed by washing the worms with three gravity steps in M9 buffer to remove residual bacteria. They were anesthetized with 25 mM Levamisole and mounted on slides with 3% agarose pads. The animals were then visualized using a Nikon SMZ18 fluorescence stereomicroscope. Quantification of fluorescence intensity was performed using ImageJ.

### Quantitative real-time PCR

*C. elegans* were grown on different bacterial diets until day 1 adulthood, 72 h after synchronized L1 larvae. For each replicate, approximately 1,000 worms were washed off the plates with M9 buffer and subjected to three gravity-washing steps to remove extracellular bacteria. These were collected in TRIzol (Invitrogen) and used for RNA isolation per the manufacturer’s instructions. The isolated RNA was treated with DNase (Thermo Fisher Scientific) to remove genomic DNA contamination (127). cDNA was synthesized from 2 μg of RNA using the RevertAid First Strand cDNA Synthesis Kit (Thermo Fisher Scientific). *In vivo* bacterial RNA was isolated along with the *C. elegans* RNA, using approximately 5,000 L4 worms and day 1 adult worms. A total of 3.5 µg of total RNA was used for cDNA synthesis using the RevertAid First Strand cDNA Synthesis Kit (Thermo Fisher Scientific). The *hilA*, *hilD*, *invA*, *ssrA*, *ssrB*, *ssaJ*, *cbiB*, *metH*, and *csgD* gene expression primer sequences are listed in Table 3. qRT-PCR was performed using PowerUp SYBR Green Master Mix (Thermo Fisher Scientific), with *ama-1* and *16S* used as the housekeeping genes for *C. elegans* and bacteria, respectively. The primer sequences are given in Table 3. The 2^−ΔΔCt^ method was used to calculate the relative gene expression.

**TABLE 3:** List of primers used in the study

Primer	Forward primer (5′−3′)	Reverse primer (5′−3′)
*ama-1*	CCCGGAGGAGATTAAACG	CATGTCATGCATCTTCCAC
*lin-3*	CTTGTCGGTGTTCTTCCC	TTCGGGAGCACTACGTTCTT
*let-23*	CCTGGAAAAGTGTTGGGA	CTGCGTCGGAAAGTGTGAAA
*mpk-1*	CCAACCACGTCAGACAACAG	ATCCCGTAAGCACCTTCTCC
*glp-1*	GCAGAAGGTTCAACGTGTGT	ATCCCGAGTCGCATACACAT
*par-5*	ATGTCCGATACCGTGGAAGA	GTTGGAAAGCTCCTGTCCCT
*ced-9*	ACGGTTGGAAATGCACAGAC	TGTTCCTTCCAGTTGTTGCG
*ced-4*	AAGTCACTCGCAATGGCTCT	CGATCCGCAACTTCATCATC
*ced-3*	GCAAGTTCAGCAAGTGTGGA	TATCCTTTGCGTGTGTCGAG
*daf-2*	CAACAGCCGTGACATTTTCAACG	GGAGATAATTTATAATTTAAGAGGC
*ins-7*	CATGCGAATCGAATACTGAA	CACTGTTTTCGAATGAAGTC
*vit-2*	CTCTCAGCAGCACATGGACA	TTGGAAGGAAGGCGTAGTCC
*vit-5*	TCTCTCTGGACTCCCAACCG	CTTGGACCACTCGGCATCTT
*rme-2*	CTTGAACTCGAGTGCCCATG	CGCAGTATTCGGCCATCAAA
*pabA KO*	GCCGCCATGATCCTGCTTATCGATA ACTATGATTCCTTTACCTGGGTGTA GGCTGGAGCTGCTTC	TCAACGCCGGAGGAAATTTTTCAGC AGAGCGTGTCCTTGTTCGCTGAGA CATATGAATATCCTCCTTAG
*pabA conf*	AAGCGAAGCTCGCGAAACTGA	CACAAATCAACGCCGGAGGAA
*cobB KO*	ATGCAGTCGCGTCGGTTTCATCGAT TAAGCCGCTTTCGTAGTGTAG GCTGGAGCTGCTTC	CGTTACCGGGCAAACAGCACTACA GCCCTTTCAGGAATCATATGAATAT CCTCCTTAG
*cobB conf*	ATGGGGATGCAGGTGGGAT	GTGAAATGTAGGCCGGATAAGG
*cobC KO*	ATGCGATTGTGGCTGGTGCGTCAT GGCGAAACCGAGGCCAATGTG TAGGCTGGAGCTGCTTC	TCACTCAGGCCGCCAGACGGCACG ACTATCATATGAATATCCTCCTTAG
*cobC conf*	GCCTCCAGCCAAAAAACGCT	TGGCTGCGGAGAATAACAG
*cobD KO*	TGGCGTTATTCAACAGCGCGCATG GCGGTAATATTCGGGAAGTGTAGG CTGGAGCTGCTTC	TCAATCAGCAGGGGCTATACCGGT AAGCACATTGCGCAGCGCCGCCAC ATATGAATATCCTCCTTAG
*cobD conf*	ATGACGCACCAGCCACAATC	CCCTGGCTTAATATCTCC
*cobS KO*	ATGAGTAAGCTGTTTTGGGCCATGC TCGCTTTTATTAGCCGCTTGCCCGT GTAGGCTGGAGCTGCTTC	TCATAACAGAGCCAGCAGAAAGATC AATTCACCAAGTTCGATCGCCGCG CCATATGAATATCCTCCTTAG
*cobS conf*	TGATATTGCCGGTCGGGTCAA	TCAATATGTTGCTGCGCACG
*cbiB KO*	ATGACGATTCTTGCCTGGTGTATCG CCTGGGTGCTGGATTTTATCATCGT GTAGGCTGGAGCTGCTTC	TCAGGCCACGCCAGATAACCCGCA CCGCGCCGCAATTAACAGCGCCAC ATATGAATATCCTCCTTAG
*cbiB conf*	GCAACACGTTTGCCAGCTATCT	GGACGCGTTTCGCGAATAAT
*metH KO*	GTGAGCAGCAAAGTTGAACAACTGCGTG CGCAGTTGTGTAGGCT GGAGCTGCTTC	TCAGTCCGCATCGTAACCCAGGTTC GGCGCGAGCCATATGAATATC CTCCTTAG
*metH conf*	TGGCAAGCTGAACATGTCTC	CGTAGAGCCTTCCTTCAGTA
*metC KO*	ATGACGGATAAACAGTTGGATACCA AACTGGTGTAGGCTGGAGCTGCTT C	TTACACAATTCTGGCGAAGCCGGCGGC TAACATATGAATATCCTCCTTAG
*metC conf*	TCATCTGTATCACACGTCGC	ACTCCTGAATCGTCCGGGAT
*metE KO*	ATGACAATATTGACTCATACCCTTG GTTTTCCTCGGTGTAGGCTGGAGC TGCTTC	TTATTTCGCCTGACGCAGATTGTGC GCCGCCATATGAATATCCTCCTTAG
*metE conf*	TTCAGTAAGCTCCTGGGGAT	AAGAGGAGATAGAGGCGCTG
16S	GCGTAGAGATCTGGAGGAAT	GTGGACTACCAGGGTATCTA
*hilA*	ATATGCCGTTCTGGTCATCC	GCCCTGTCCGTACAGTGTTT
*hilD*	CCTGCCAGAAGAGAGGTATT	CAGTAAGCAGGACAGCAGA
*invA*	CGTTTTGCTTCACGGAATTT	AGTGCCGGTTTTATCGTGAC
*ssrA*	TGCACCGCTGAGCACACGTT	ATTGCACCGAGTACGCCATT
*ssrB*	ATCATTAACGGCATTATGAA	GTATGCTGTGTAAACCAGAA
*ssaJ*	CCGTTGTAAACTGCCGATGC	ACGGCATGTGATGTGGATCT
*csgD*	GCCTCATATTAACGGCGTGT	GCGTAATCAGGTAACTGGCT
*cbiB RT*	ATTGTGCGTCGCTATTGTCC	ATCATCCAGACTTCGACGCT
*metH RT*	GGCGGTATGGGCACTATGAT	AGTAGGCGTTGTGGATAGCG

### Quantification of cytosolic reactive oxygen species levels

ROS levels were measured using 2′,7′-dichlorofluorescein diacetate (DCFHDA) (Sigma-Aldrich #35845) (128). Before the experiments, a fresh 50 μM working solution was prepared from a 100 μM stock solution in M9 buffer. Synchronized L1 larvae were grown on different bacterial diets until day 1 of adulthood at 20°C. About 100 worms were transferred to 300 μL of dye (150 μL of 50 μM DCFHDA working solution and 150 μL of M9 buffer), giving a final concentration of 25 μM. The samples were incubated in the dark at room temperature for 3 h with gentle shaking. Worms were then settled by gravity, the supernatant was removed, and the samples were washed three times with PBS plus 0.01% Triton X-100. DCF fluorescence was imaged using a GFP filter on a fluorescence microscope, and at least ten worms per condition and three biological replicates were analyzed.

### Quantification of bacterial burden in the *C. elegans* intestine

The intestinal WT-STM, Δ*cbiB*, and Δ*metH* bacterial loads were quantified by measuring colony-forming units (CFU) as described earlier (40, 41). In brief, at the D-1 adult stage, infected worms were transferred from the NGM infection plates to fresh *E. coli* OP50 plates for 10 min to eliminate bacteria stuck to their bodies. Subsequently, 20 *C. elegans* per condition were transferred into a 1 mL antibiotic cocktail (25 mM Levamisole, 50 µg/mL of Kanamycin, 50 µg/mL of Ampicillin, 50 µg/mL of Chloramphenicol, M9 buffer), incubated for 10 min, washed thrice with the cocktail, and 50 μL from the last wash was plated to monitor the extracellular bacterial load. The remaining supernatant was removed; 50 μL of 0.01% Triton X-100 was added to the pellet, and the mixture was incubated for 10 min; then lysed with a pestle. A total of 50 μL of 0.01% Triton X-100 was added, and from it, 50 μL was taken for serial dilutions of the lysates (10^3^ and 10^4^) and plated on SS agar plates for WT-STM, while Δ*cbiB* and Δ*metH* were plated on LB plates containing 50 µg/mL of Kanamycin and grown overnight at 37°C. Single colonies were counted the next day and expressed as CFU per animal.

### *C. elegans* lysate preparation for vitamin B12 isolation

The *C. elegans* lysate preparation for B12 isolation was performed as described by reference 23 with slight modifications. Approximately 5,000 day 1 adult *C. elegans* were washed off the NGM plates seeded with *E. coli* OP50, WT-STM, *E. coli* OP50 supplemented with vitamin B12, Δ*cbiB*, and Δ*metH* using M9 buffer and subjected to gravity-settling washing steps to remove extracellular bacteria. The worm pellets were washed 3–4 times with M9 buffer to ensure the thorough elimination of any residual bacterial food lawn. The wet weights of the *C. elegans* were recorded, and the cleaned worm pellets were flash-frozen in liquid nitrogen before being stored in a −80°C deep freezer for 15 min. Following this, the frozen *C. elegans* pellets were resuspended in 0.1 M sodium acetate buffer (pH 5.2). Given that cobalamin exists in several unstable intracellular forms (methylcobalamin, adenosylcobalamin, and hydroxocobalamin), extraction assays typically incorporate a small trace amount of cyanide to reliably convert all variants into a uniform compound: cyanocobalamin. Accordingly, 0.01% sodium cyanide (NaCN) was added to the sodium acetate buffer. The *C. elegans* were then kept in the −80°C deep freezer for 15 min. After freezing, the *C. elegans* pellets were crushed with a pestle and stored at −80°C for another 15 min before thawing on ice. The lysates were incubated at 100°C on a heat block for 15 min in the dark. The acetate buffer at pH 5.2 helps prevent vitamin B12 degradation, while heat facilitates the dissociation of B12 from *C. elegans* protein. Immediately after incubation, the tubes were plunged into ice for 5 min to rapidly cool and precipitate the proteins. The tubes were then centrifuged at 14,000 RPM for 15 min at 4°C. The clear, translucent supernatants were collected, and the pellets containing denatured host-cell debris were discarded. Finally, the free, concentrated vitamin B12 in the supernatant was used to estimate B12 via a microbiological assay.

### Preparation of the Δ*metE* bioassay inoculum

As previously described (63), with slight modifications, the *Salmonella* Δ*metE* mutant strain was grown overnight in LB broth with kanamycin (50 µg/mL) at 37°C. The bacterial cells were centrifuged at 5,000 RPM for 5 min at room temperature, and the supernatant was discarded. The pellets were washed four times with pure, sterile M9 minimal salts (Na_2_HPO_4_, KH_2_PO_4_, NaCl, NH_4_Cl) to completely starve out any remaining intracellular or environmental methionine. The washed pellets were resuspended in 1 mL of sterile M9 minimal medium (1× M9 minimal salts, 0.4% glucose, 2 mM MgSO_4_, 0.1 mM CaCl_2_, 50 µg/mL Kanamycin), and the solution was diluted until the OD600 reached 0.2 to prepare the working inoculum.

### Preparation of the B12 standard curve and test samples

A serial 1:2 dilution gradient was established using a pure vitamin B12 (Cyanocobalamin, Sigma V2876) reference standard, which was diluted in sterile M9 medium. The target concentration ranged from 0 to 1,000 pM, and the dilutions were prepared in a 1.5 mL microcentrifuge tube, in the dark. The bacterial inoculum, which exhibited an OD600 of 0.2, was diluted approximately 1:10 by adding 67 µL of inoculum to 600 µL of M9 medium containing serially diluted vitamin B12, resulting in a final OD600 of 0.02. A 200 µL volume of this mixture was subsequently transferred into a sterile, clear-bottom 96-well microplate in triplicate. For the test samples, 100 µL of lysate was diluted with 900 µL of M9 medium and syringe-filtered in a laminar-flow hood. These samples were then subjected to twofold serial dilutions. The plates were incubated at 37°C for 24 h with orbital shaking at 230 RPM. The absorbance at OD600 was measured using an Agilent microplate reader and plotted. The concentration of the diluted sample extract *C*_diluted_, expressed in (pM), loaded into the bioassay wells was calculated via single-point standard calibration by determining the ratio of the sample’s integrated growth area against a known reference standard, as defined:


$$C\left(diluted\right)=\frac{AUC\left(sample\right)}{AUC\left(standard\right)}\;\times \;C\left(standard\right)$$

Where AUC(sample) is the integrated area under the kinetic growth curve for the diluted worm extract, AUC(standard) is the area under the growth curve for the reference standard, and *C*(standard) is the known concentration of the reference standard (pM).

The absolute concentration of the original, undiluted sample stock extract, *C*(extract), was back-calculated using the respective dilution factor as,


C(extract)=C(diluted)×Dilution factor 

The total cellular vitamin B12 content normalized to the initial tissue mass was subsequently determined using the formula,


$$Vitamin\;B12\;content\;=\frac{C\left(extract\right)\times V\left(extract\right)}{Wet\;weight}$$

### Lifespan assay

For lifespan experiments, approximately 100 age-synchronized L1 larvae were transferred to NGM plates seeded with *E. coli* OP50, wild-type *Salmonella*, SPI-1 effector mutants, such as Δ*hilA*, Δ*sipA*, Δ*sipD*, Δ*sopA*, and Δ*sopB*, and *ΔcbiB*, and Δ*metH*, *E. coli* OP50 supplemented with either vitamin B12 or methionine, respectively, and grown up to late L4 larval stages, i.e., 48 h post-L1 stage. A total of 30–40 late L4 larvae were then transferred to NGM plates supplemented with 50 µg/mL of 5-fluoro-2′-deoxyuridine at 20°C, seeded with the corresponding bacterial strains. Worms were monitored for mortality after 24 h and then every other day. Animals that did not respond to touch were scored as dead, with at least three biological replicates per condition.

### Mitochondrial DNA quantification

As previously described, mtDNA was quantified by a quantitative real-time PCR-based method (129, 130). Briefly, 2-day-old adult hermaphrodites were singly picked into 0.2 mL PCR tubes containing 20 µL worm lysis buffer (50 mM KCl, 10 mM Tris, pH 8.4, 25 mM MgCl_2_, 0.45% Tween 20, 60 µg/mL proteinase K). After freezing at −80°C for 10 min, the tubes were incubated on the worm lysis program in a thermocycler at 60°C for 1 h, 95°C for 15 min, and then 4°C for 5 min. The 5′-GTTTATGCTGCTGTAGCGTG-3′ and 5′-CTGTTAAAGCAAGTGGACGAG-3′ primer pairs were used for measuring mtDNA levels. These primers hybridize to the *atp-6* gene in the mitochondrial genome (mtDNA). The results were normalized to genomic DNA amplified with the 5′-TGGAACTCTGGAGTCACACC-3′ and 5′-CATCCTCCTTCATTGAACGG-3′ primer pair, which hybridizes to the *ama-1* gene. Quantitative PCR was performed using PowerUp SYBR Green master mix (Thermo Fisher Scientific) on a Bio-Rad CFX96 real-time PCR system, and the experiment was repeated three times. The 2^−ΔΔCt^ method was used to calculate the relative gene expression.

### Quantification and statistical analysis

Statistical analysis was performed using Prism 10 (GraphPad). All error bars represent the mean ± standard deviation (SD). For comparisons involving more than two samples, an ordinary one-way ANOVA was conducted, followed by Tukey’s multiple comparisons test. In the figures, asterisks (*) signify statistical significance as follows: *P < 0.05; **P < 0.01; ***P < 0.001; ****P < 0.0001, relative to the appropriate controls. For experimental comparisons involving exactly two groups or conditions, statistical significance was determined using a two-tailed, unpaired Student’s *t*-test, where *P < 0.05; **P < 0.01*.* The Kaplan-Meier method was used to calculate survival fractions, and statistical significance between survival curves was determined using the log-rank test. All experiments were conducted at least three times.

## Data Availability

All the data supporting the findings of this study are provided within the article and its Supplemental material. Additional information related to this study is available from the corresponding author upon request.

## SUPPLEMENTAL MATERIAL

Supplemental figure legends spectrum.00827-26-s0001.docx Legends for Fig. S1 to S5.

Fig. S1 spectrum.00827-26-s0002.tif Gene expression related to various signaling pathways of infected *C. elegans*.

Fig. S2 spectrum.00827-26-s0003.tiff Optimization of the Salmonella ∆metE microbiological bioassay across varying vitamin B12 standard concentrations.

Fig. S3 spectrum.00827-26-s0004.tif *Salmonella* Typhimurium accelerates development through vitamin B12-dependent metabolism in *C. elegans*.

Fig. S4 spectrum.00827-26-s0005.tiff Evaluation of vitellogenin synthesis and reproductive capacity in *C. elegans* across different bacterial diets.

Fig. S5 spectrum.00827-26-s0006.tiff Representative fluorescence images and (B) quantification of VIT-2::GFP expression.

ASM does not own the copyrights to Supplemental Material that may be linked to, or accessed through, an article. The authors have granted ASM a non-exclusive, world-wide license to publish the Supplemental Material files. Please contact the corresponding author directly for reuse.
