# Supplementary material for: Salmonella Typhimurium vitamin B12-dependent methionine metabolism regulates Caenorhabditis elegans development and vitellogenesis
Source: Microbiol Spectr. 2026 Sep 29;14(11):e00827-26. doi: 10.1128/spectrum.00827-26 (PMC13729190; doi:10.1128/spectrum.00827-26)
Supplement: Supplementary file 1 [file spectrum.00827-26-s0001.docx]

**Supplementary figure legends**

Fig. S1 Gene expression related to various signaling pathways of *C. elegans* exposed to *E. coli* OP50, WT-STM*, ∆hilA*, *∆avrA*, *∆sptP*, *∆sipD*, *∆ssaV*, *∆ygiM*, *∆rspA*, *∆sopB*, *∆mgtC*, *∆phoP*, and *∆fepB.* (A) Genes such as *lin-3, let-23, mpk-1, par-5, glp-*1 are involved in the RAS/MAPK signaling pathway during oogenesis, (B) *daf-2* and *ins-7* are involved in the Insulin/IGF-1 signaling pathway, and (C) *ced-9, ced-4,* and *ced-3* are involved in the programmed-cell death pathway. An ordinary one-way ANOVA, post-hoc Tukey HSD test was performed. *****P<0.0001*; ****P < 0.001; **P < 0.01; * P < 0.05.* (D) Representative Kaplan-Meier plot comparing survival of N2 worms exposed to the indicated bacterial conditions.

Fig. S2 **Optimization of the** Salmonella *∆*metE **microbiological bioassay across varying vitamin B12 standard concentrations and dilutions of *C. elegans* extracts from indicated bacterial conditions.** **(A-C)** Growth kinetics of the vitamin B12/methionine auxotroph Salmonella *∆*metE were monitored via OD600 over a 24-hour period to evaluate assay saturation thresholds and resolution dynamics. The performance of worm tissue extract under various bacterial conditions was evaluated at three standard concentrations and their dilutions.

Fig. S3 *Salmonella* Typhimurium accelerates development through vitamin B12-dependent metabolism in *C. elegans.* (A) Represents the quantification of embryos and (B) Oocyte production in *mmcm-1* animals on the indicated bacterial conditions. An ordinary one-way ANOVA, post-hoc Tukey HSD test was performed. *****P<0.0001*; ****P < 0.001; **P < 0.01; * P < 0.05.*

Fig. S4 Evaluation of vitellogenin synthesis and reproductive capacity in C. elegans across different bacterial diets. **(A, B)** Characterization of intestinal vitellogenin expression using a transgenic reporter strain. **(A)** Representative fluorescence images of adult C. elegans expressing a **VIT-2::GFP** reporter after feeding on indicated bacterial conditions, including E. coli OP50, E. coli OP50 supplemented with vitamin B12 or methionine, respectively, Comamonas aquatica (DA), WT-STM, Δ*cbiB,* and Δ*met.* **(B)** Quantification of relative intestinal **VIT-2::GFP fluorescence intensity. (C)** Total **number of embryos per worm** inside the uterus and **(D)** total **number of oocytes per gonad arm in N2 worms under the** indicated bacterial conditions. **(E)** Total **number of embryos per worm** inside the uterus and **(F)** total **number of oocytes per gonad arm in *metr-1* worms under the** indicated bacterial conditions. An ordinary one-way ANOVA, post-hoc Tukey HSD test was performed. *****P<0.0001*; ****P < 0.001; **P < 0.01; * P < 0.05.* Scale bar = 100 μm.

Fig. S5 (A) Representative fluorescence images and (B) Quantification of VIT-2::GFP expression in *vit-2::gfp* transgenic worms exposed to *E. coli* OP50, wildtype *Salmonella, E. coli* OP50 supplemented with either vitamin B12 or methionine, respectively, for 24 hours from L4 onwards. GFP levels were increased in the day-1 worms exposed to *E. coli* OP50 supplemented with vitamin B12 and methionine, respectively. (C) Representative fluorescence images and (D) Quantification of GST-4::GFP expression in *gst-4::gfp* transgenic worms exposed to *E. coli* OP50, wildtype *Salmonella, E. coli* OP50 supplemented with either vitamin B12 or methionine, respectively, for 24 hours from L4 onwards. The worms exposed to *E. coli* OP50 supplemented with vitamin B12 and methionine, respectively, show decreased GFP expression compared to *E. coli* OP50, while those exposed to STM show GFP expression comparable to *E. coli* OP50. An ordinary one-way ANOVA, post-hoc Tukey HSD test was performed. *****P<0.0001*; ****P < 0.001; **P < 0.01; * P < 0.05.* (E) Representative Kaplan-Meier plot comparing survival of N2 worms exposed to *E. coli* OP50, wildtype *Salmonella,* Δ*cbiB,* Δ*metH, E. coli* OP50 supplemented with either vitamin B12 or methionine, respectively. Scale bar = 100 μm.
